# The Src family kinase YES regulates DNAJB6b chaperone activity to modulate tau aggregation in Alzheimer’s disease

**DOI:** 10.1016/j.jbc.2026.113363

**Published:** 2026-07-24

**Authors:** Meng-Chieh Lin, Huan-Yun Chen, Te-Sheng Lin, Sue-Jane Lin, Pei-Jie Lin, Chia-Yu Liao, Nei-Li Chan, Chin-Hsien Lin, Shu-Chun Teng

**Affiliations:** 1 Department of Microbiology, College of Medicine, National Taiwan University, Taipei, Taiwan; 2 Department of Neurology, National Taiwan University Hospital, Taipei, Taiwan; 3 Institute of Biochemistry and Molecular Biology, College of Medicine, National Taiwan University, Taipei, Taiwan; 4 Center for Precision Medicine, National Taiwan University, Taipei, Taiwan

**Keywords:** Alzheimer’s disease, co-chaperone, DNAJB6b, J-domain proteins, Src family kinases, tau, YES

## Abstract

Alzheimer's disease (AD) is a neurodegenerative disorder characterized by a gradual deterioration of memory. While its prevalence increases with age, the underlying mechanisms remain incompletely understood. Here, we examine the biological consequences of phosphorylation-mediated chaperone activity in AD-associated tau aggregates. Increased phosphorylation of DNAJB6b at Y53 is observed in the brain lysates of AD patients. Our research found that an activated Src family kinase, YES, phosphorylates Y53 in the J-domain of DNAJB6b and enhances the binding between DNAJB6b and Hsp70. The strengthened association between DNAJB6b and Hsp70 may lead to the accumulation of tau aggregates. These findings suggest that up-regulation of YES kinase modifies DNAJB6b and that the resulting alteration in DNAJB6b-dependent tau disaggregation may contribute to an increased risk of AD.

Alzheimer's disease (AD), a common neurodegenerative disease, is characterized by cognitive impairment and memory decline (1). The pathological hallmarks of aging-related neurodegenerative disease (2) are the loss of proteostasis and the accumulation of toxic aggregates. Specific abnormal AD structures include intracellular aggregates of hyperphosphorylated tau protein and extracellular amyloid-β plaques in the hippocampus (HIP) (3). Tau protein, a member of the microtubule-associated protein family, normally facilitates microtubule assembly and stabilizes microtubule dynamics. However, abnormal folding of the tau protein can increase its propensity for self-aggregation, leading to the formation of intracellular neurofibrillary tangles, which are the primary pathological hallmarks of AD and cause cognitive decline. Aggregated insoluble tau protein in cells impairs a broad range of cellular processes, including axonal transport (4), pre and prosynapse transmission (5), and mitochondrial function (6), collectively leading to profound damage to neuronal health. Additionally, misfolded tau protein ‘spreads’ its toxicity to other native tau molecules and induces aberrant conformational changes (7). The pathological progression of AD can be classified into six stages, proposed by Braak (8), based on the distribution pattern of neurofibrillary change. The progression begins with the formation of early-stage intraneuronal tau aggregates in the transentorhinal cortex and culminates in advanced isocortical degeneration driven by high-density neurofibrillary tangles. This hierarchical spread of tau pathology correlates with subtle memory impairments, sequential cognitive decline, and advancing to severe cortical dysfunction.

The heat shock protein 70 (Hsp70) system is constitutively active and critical for maintaining cellular proteostasis by promoting proper protein folding and directing misfolded or aggregation-prone proteins toward degradation (9, 10). Activating a Hsp70 reaction machinery cycle requires the active participation of Hsp70s, cochaperone J-domain proteins (JDPs), nucleotide exchange factors, and ATP-ADP exchange (10, 11). Human Hsp70 comprises 13 compartment-specific isoforms that fulfill organelle-specific functions. In the cytoplasm, it includes stress-inducible HSPA1A/HSP70 and constitutively expressed HSPA8/HSC70 (12, 13). Hsp70 exhibits intrinsically low ATPase activity in its unliganded state and requires JDPs for functional activation (14). JDPs perform dual roles: delivering substrates to Hsp70’s substrate-binding domain and allosterically stimulating ATP hydrolysis through conserved J-domain interaction. This ATPase-mediated activation induces structural transitions in Hsp70, thereby generating mechanical forces that drive substrate folding. The coordinated JDP-mediated substrate refolding and NEF-driven nucleotide exchange establish an iterative folding mechanism, demonstrating an essential partnership in proteostasis. This ATP-dependent cycle highlights how JDPs critically regulate substrate and ATPase activation, ensuring efficient protein quality control.

Over 50 JDPs found in humans can be classified into 3 subfamilies: DNAJA, DNAJB, and DNAJC (15). Many findings suggest that the DNAJB subfamily is associated with the delivery of distinct types of misfolded clients. DNAJB1, for example, has been shown to slow the spread of α-synuclein aggregation associated with Parkinson’s disease (16, 17). Misfolding-prone A25T mutant transthyretin and amyloid precursor protein can be bound to secrete DNAJB11 to prevent extracellular aggregation and proteotoxicity during ER stress (18). Among these, DNAJB6 has emerged as a particularly potent suppressor of amyloidogenesis. It is ubiquitously expressed across various tissues, including the brain and muscle, and exists in two alternative splicing isoforms: the nuclear-localized DNAJB6a (36 kDa) and cytoplasmic-localized DNAJB6b (27 kDa) (19). While DNAJB6a effectively prevents nuclear polyglutamine aggregation found in Huntington’s disease (20), the cytoplasmic DNAJB6b is critical for managing aggregates associated with tauopathies and myopathies. Previous studies have shown that reduced DNAJB6b expression is associated with increased tau aggregate load in neuroblastoma cells (21) and with age-dependent tau accumulation in rTg4510 mouse models (21). Besides, DNAJB6b is the primary isoform implicated in Limb-Girdle Muscular Dystrophy Type D1 (LGMDD1). Dominant missense mutations within the glycine/phenylalanine (G/F) domain of DNAJB6b—such as F89I, F93L, and P96R—disrupt its function at the Z-disc of muscle sarcomeres, leading to myofibrillar protein aggregation and vacuolar myopathy (22, 23). Structurally, like other DNAJB subfamilies, DNAJB6 contains an N-terminal J-domain followed by a G/F-rich, a serine/threonine-rich, and a functionally unclear C-terminal domain (24). The J-domain is a signature domain of JDPs, containing a His-Pro-Asp motif required for direct interaction with Hsp70 (25). Recent structural studies suggest that the G/F-rich region may act as an autoinhibitory switch (26), while the serine/threonine-rich region drives the oligomerization necessary to recognize and suppress toxic protein aggregates (27). Despite the importance of the JDP-Hsp70 system in maintaining cellular proteostasis networks and the well-known structural linkage between mutations in DNAJB6b and LGMDD1 disease, the current understanding of how posttranslational modifications (PTMs) of JDP regulate the chaperone system remains limited.

Over 90% of ADs are not caused by genetic mutations (28), suggesting that environmental, post-transcriptional, and post-translational factors during the aging process may be the key regulators. Hundreds of PTMs on chaperones/co-chaperones, known as chaperone codes, are thought to alter chaperone activity and protein interactions (29); however, our understanding of the functional roles of these codes remains limited (17, 30, 31). Combined with the existing knowledge of the specific association between DNAJB6b and tau protein, here we explore the influence of phosphorylation on regulating the co-chaperone activity of DNAJB6b.

## Results

### Dephosphorylation of DNAJB6b on tyrosine 53 enhances its ability to prevent tau aggregation in SH-SY5Y neuroblastoma cells

**Figure 1:**
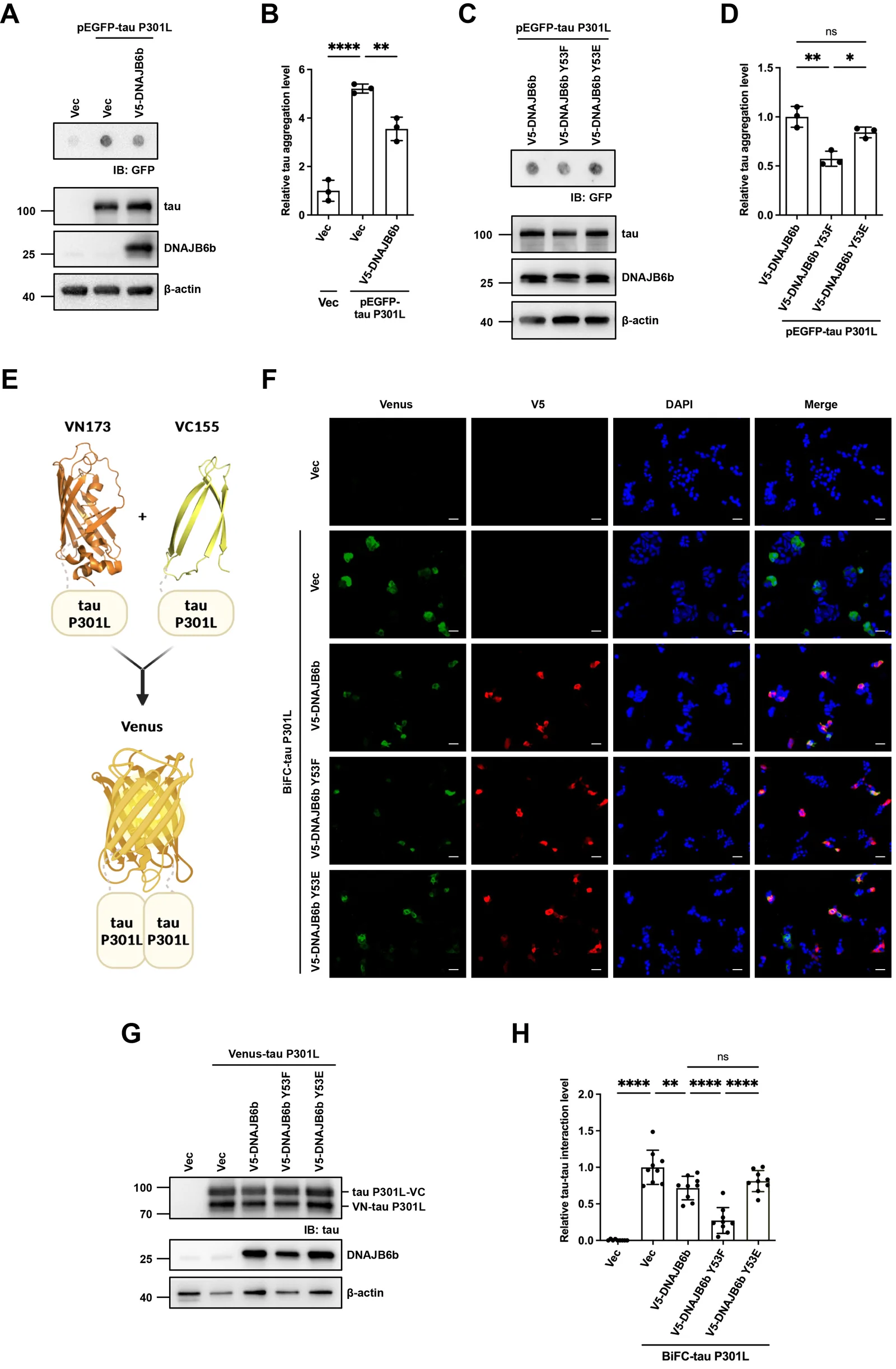
**Dephosphorylation of DNAJB6b enhances its ability to prevent tau aggregation.***A*, the filter trap assay for analysis and quantification of aggregated tau proteins. Retained proteins were immunoblotted using an anti-GFP antibody to detect tagged tau. *B*, β-actin served as a loading control for quantification of aggregated tau proteins in (*A*) (*n* = 3). *C*, SH-SY5Y cells co-transfected with EGFP-tau P301L and different combinations of the V5-DNAJB6b Y53 constructs for 48 h using the same filter trap assay method as in (*A*). *D*, β-actin was used as a loading control for quantification of aggregated tau proteins in (*C*) (*n* = 3). *E*, schematic representation of BiFC maturation for visualization of tau aggregation. Nonfluorescent N-terminal (VN173) and C-terminal (VC155) compartments of Venus protein fused to full-length tau P301L. Aggregation of the tau protein leads to fluorescence emission. *F*, Representative fluorescent images of the BiFC assay. SH-SY5Y cells were transfected with VN-tau P301L and tau P301L-VC (BiFC-tau P301L), along with different V5-DNAJB6b Y53 constructs. The fluorescence emitted by Venus complementation was recorded 48 h after transfection. Cells were immunostained with V5 antibody, and nuclei were counterstained with DAPI. Representative images were taken at 10× magnification. The scale bar represents 20 μm. *G*, immunoblotting with anti-tau and anti-DNAJB6 antibodies indicated the expression levels of BiFC-tau P301L and DNAJB6b in cells, respectively. *H*, quantification of BiFC-fluorescence of (*F*) (*n* = 3). The percentage of fluorescent (BiFC^+^) SH-SY5Y cells of the total number of cells (DAPI^+^) was quantified in at least 3 random-field images taken at 10x magnification. Data in (*B*, *D–H*) were represented as mean ± SD, and *p*-values were calculated *via* one-way ANOVA followed by Dunnett's test. (ns, not significant; ∗*p* < 0.05, ∗∗*p* < 0.01, ∗∗∗∗*p* < 0.0001). BiFC, bimolecular fluorescence complementation.

Previous studies have shown that co-chaperone DNAJB6b is associated with the aggregate load of tau in neuroblastoma cells (21). To determine the functional role of the chaperone code on DNAJB6b, we considered candidates based on available data from PhosphositePlus (32) (see Table S1) and PhosphoNET (see Table S2). Since the J-domain of DNAJB6b is crucial for preventing tau aggregation, phosphorylation of tyrosine 53 (Y53), identified as the most referenced site on the J domain through proteomic mass spectrometry, was selected for further investigation. To evaluate the effect of the phosphomimetic substitutions in cells, the Y53 site on DNAJB6b is replaced by phenylalanine (F) (nonphosphorylatable variants) or glutamic acid (E) (pseudo-phosphorylated mutants). The filter trap assay was first used to detect and quantify protein aggregation levels. Human neuroblastoma SH-SY5Y cells overexpressing the disease-associated EGFP-tagged tau P301L exhibited a significant tau aggregation level. However, with co-overexpression of V5-tagged WT DNAJB6b, tau aggregates were reduced (Fig. 1, *A* and *B*), consistent with previous observations (21). To further determine whether the phosphorylation of DNAJB6b Y53 affects its ability to reduce tau aggregation, we co-overexpressed EGFP-tau P301L with either non-phosphorylatable V5-DNAJB6b Y53F or pseudo-phosphorylated V5-DNAJB6b Y53E in SH-SY5Y cells. A decrease in aggregated tau was observed in the V5-DNAJB6b Y53F mutant group. In contrast, the V5-DNAJB6b Y53E mutant displayed no significant difference compared to that in the V5-DNAJB6b WT group (Fig. 1, *C* and *D*), suggesting that dephosphorylation of Y53 may strengthen the function of DNAJB6b, leading to reduced accumulation of insoluble tau.

To confirm whether the dephosphorylation of DNAJB6b Y53 enhances co-chaperone function to prevent tau aggregation *in vivo*, a tau bimolecular fluorescence complementation (BiFC) assay was performed to evaluate the tau-tau interaction level (33, 34) (Fig. 1, *E*–*H*). Nonfluorescent N-terminal (1–172 a.a., VN173) and C-terminal (155–238 a.a., VC155) compartments of Venus protein fused to full-length tau P301L were co-overexpressed equally with V5-DNAJB6b WT, Y53F, or Y53E in SH-SY5Y cells (Fig. 1*G*). Venus fluorescence protein complex can be formed at close distances (less than 10 nm) as tau assembles in cells (tau-BiFC signal) to visualize protein-protein interactions (Fig. 1*E*). As expected, overexpression of the V5-DNAJB6b WT showed a decrease in the tau-BiFC signal compared to the Venus-tau P301L-only group (Fig. 1*F*). Additionally, the V5-DNAJB6b Y53F-overexpressed group exhibited a significantly lower tau-BiFC positive signal than both the V5-DNAJB6b WT and Y53E mutants, indicating a reduced level of tau-tau interaction (Fig. 1*H*). Conversely, a similar tau-BiFC positive signal was detected in the overexpression of V5-DNAJB6b WT and Y53E. Together, these results suggest that dephosphorylation of DNAJB6b Y53 may enhance the co-chaperone activity of DNAJB6b in resolving tau aggregation.

### DNAJB6b Y53 phosphorylation is correlated with AD

**Figure 2:**
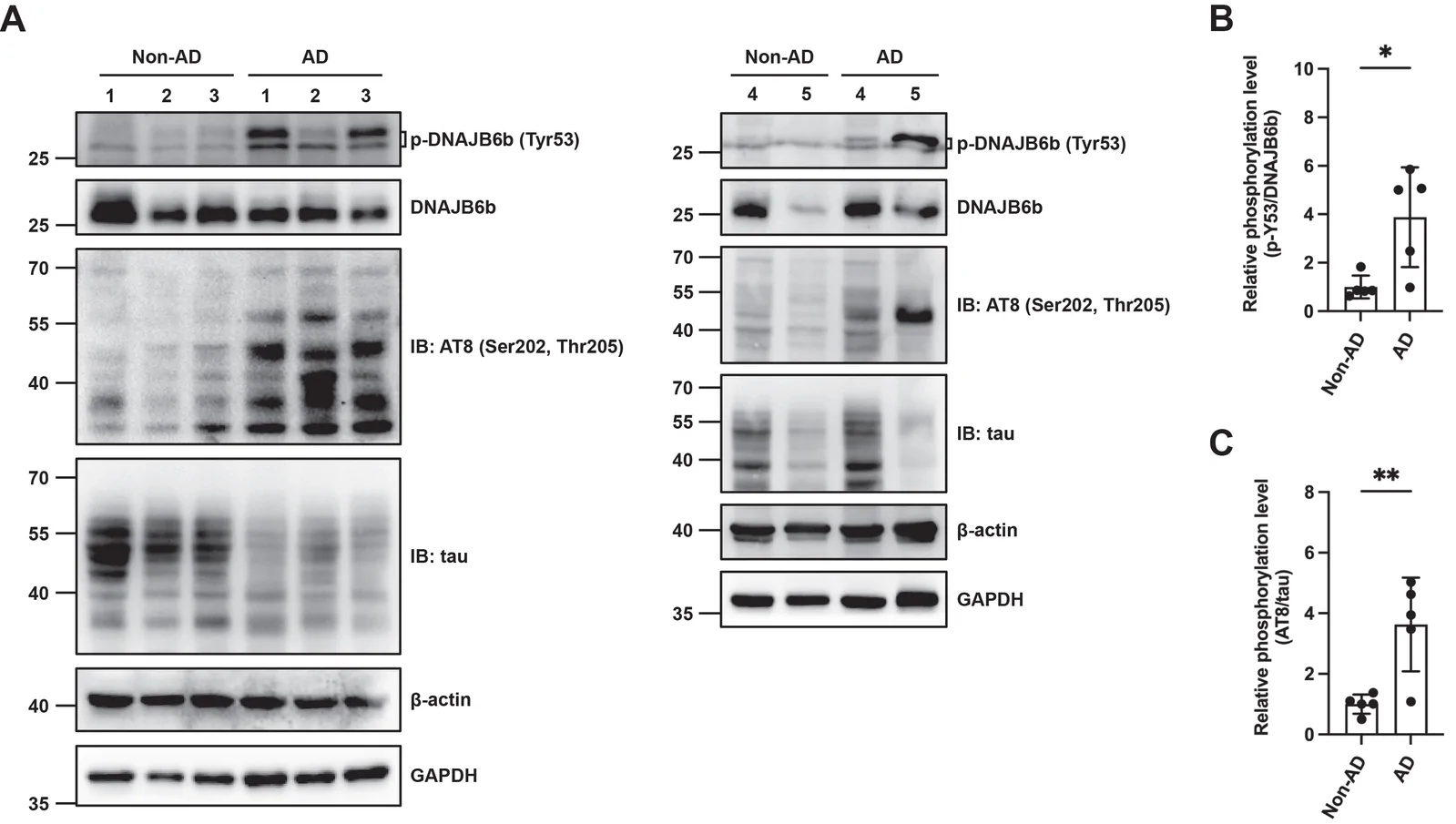
**DNAJB6b Y53 phosphorylation is increased in AD brain tissues.***A*, commercial human whole brain lysates were acquired, and the levels of each protein were analyzed using western blotting with the specified antibodies. Phosphorylation of tau at Ser202 and Thr205 was detected using an AT8 antibody. β-actin and GAPDH served as a loading control. The catalog numbers for the non-AD brain lysates from one to five are #C511134, #822400674, #C807591, #C910514, and #822600376. The catalog numbers for the AD brain lysates from one to five are #C511136, #C511137, #822304853, #C910505, and #822502057. *B*, quantification of the ratio of phosphorylation levels of DNAJB6b Y53 compared with those of the endogenous DNAJB6b in (*A*) (*n* = 5). *C*, quantification of p-tau (Ser202 and Thr205) to tau in (*A*) (*n* = 5). Data in (*B* and *C*) were expressed as mean ± SD, and *p*-values were calculated *via* unpaired two-tailed Student’s *t* test. (∗*p* < 0.05, ∗∗*p* < 0.01).

Next, we examined whether phosphorylation of DNAJB6b Y53 exists in AD brains. Phosphor-Y53 antibodies were generated by immunizing a BALB/c mouse with the synthesized DNAJB6b phosphor peptide (AEApYEVLSDAKKC). The antibody specificity was tested and confirmed using the filter trap assay and Western blotting (see Fig. S1). Five non-AD and five AD human brain lysates were then assessed for the phosphorylation level of DNAJB6b Y53 by Western blot analysis (Fig. 2*A*). The phosphorylation of DNAJB6b Y53 was significantly increased in AD brains compared with non-AD brains (Fig. 2*B*). The phosphorylation-specific monoclonal antibody AT8 is used to detect phosphorylation of serine 202 and threonine 205 in neuronal tau, which drives its aggregation. Quantification of the immunoblots revealed higher tau phosphorylation in AD brains (Fig. 2*C*), which paralleled the increase in DNAJB6b Y53 phosphorylation.

A further experiment was then conducted to evaluate whether tau expression *per se* causes DNAJB6b to modify its activity through posttranslational modification. The Y53 phosphorylation level is evaluated in SH-SY5Y cells overexpressing EGFP-tau WT and P301L mutant. As shown in Fig. S2*A*, the EGFP-tau P301L-overexpressing group exhibited a significantly lower Y53 phosphorylation level. On the other hand, the EGFP-tau WT-overexpressed group showed no differences from the control. Together, the results suggest that DNAJB6b may become hypo-phosphorylated, leading to its hyperactivation, to deal with the overwhelming aggregation-prone tau proteins overexpressed in the cells.

### SFKs regulate Y53 phosphorylation on DNAJB6b in SH-SY5Y cells

**Figure 3:**
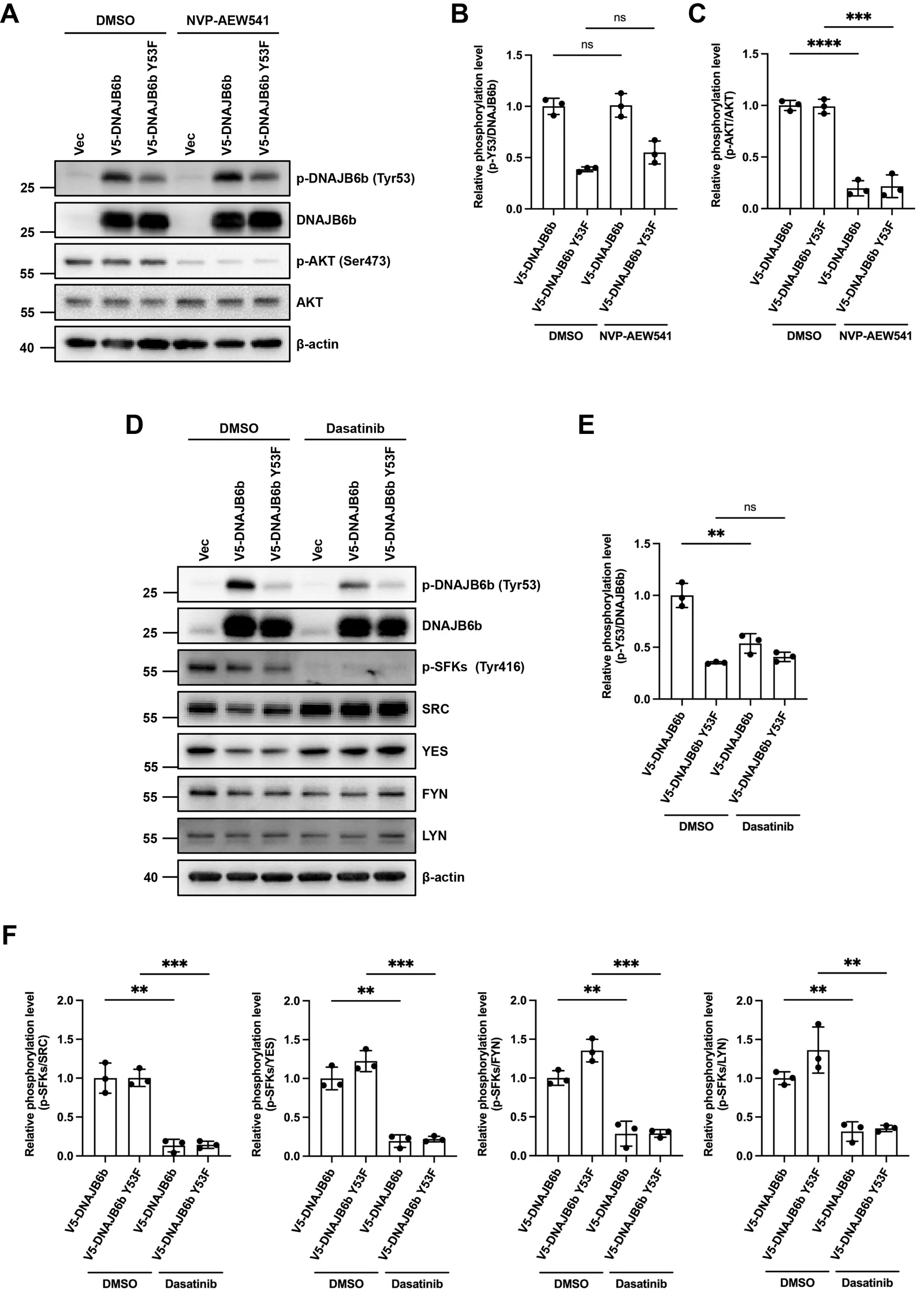
**SFKs mediate the phosphorylation of DNAJB6b at Y53.***A*, phosphorylation of DNAJB6b at the Y53 site was evaluated in NVP-AEW541-treated SH-SY5Y cells by western blotting. Cells were over-expressed with empty vector (pcDNA/FRT/TO-V5) and indicated V5-DNAJB6b constructs for 48 h, followed by NVP-AEW541 treatment or solvent control DMSO for 2 h. Proteins were analyzed by immunoblotting using the indicated antibodies. *B and C*, quantification of the ratio of p-DNAJB6b Y53 to DNAJB6b WT or DNAJB6b Y53F (*B*) and the ratio of p-AKT Ser473 to AKT (*C*) in (*A*) (*n* = 3). *D*, the phosphorylation level of DNAJB6b Y53 was evaluated in dasatinib-treated cells by western blotting. SH-SY5Y cells were overexpressing empty vector (pcDNA/FRT/TO-V5) and indicated V5-DNAJB6b constructs for 48 h, followed by dasatinib treatment or DMSO solvent control for 2 h. Proteins were analyzed by immunoblotting using the indicated antibodies. *E and F*, quantification of the ratio of p-DNAJB6b Y53 to DNAJB6b WT or DNAJB6b Y53F (*E*) and the ratio of p-SFKs Tyr416 to SRC, YES, FYN, or LYN (*F*) in (*D*) (*n* = 3). Data in (*B* and *C*, *E and F*) were expressed as mean ± SD, and *p*-values were calculated *via* unpaired two-tailed Student’s *t* test. (ns, not significant; ∗∗*p* < 0.01, ∗∗∗*p* < 0.001, ∗∗∗∗*p* < 0.0001).

To further investigate the possible kinase responsible for DNAJB6b Y53 phosphorylation, we employed the NetPhos 3.1 server (35, 36) to predict the top 3 candidates, including insulin receptor (INSR) kinase, SRC, and epidermal growth factor receptor kinase (see Table S3). The respective final concentrations of kinase inhibitors were derived from the half-maximal inhibitory concentration ($IC_{50}$) (see Fig. S3). After cells were treated with the kinase inhibitors, we examined the phosphorylation levels of DNAJB6b Y53 and the kinase activity, compared with the solvent control. First, V5-DNAJB6b-overexpressing cells were incubated with 10 μM NVP-AEW 541, a specific inhibitor of INSR kinase (Fig. 3, *A*–*C*). The suppression of AKT serine 473 phosphorylation served as an indicator of kinase activity (Fig. 3*C*) (37, 38). The results indicated no significant difference in p-Y53 level between the WT group treated with and without NVP-AEW 541 (Fig. 3*B*), suggesting that INSR kinase is not responsible for the phosphorylation of DNAJB6b at Y53.

Next, we examined the second candidate; SFKs. Human SFKs comprise 11 members and can be categorized into 3 subfamilies based on their homology (39, 40). The SRC subfamily (SRC, FYN, YES, and FGR) and the LYN subfamily (LYN, HCK, LCK, and BLK) exhibit similar modes of regulation of cellular viability, proliferation, differentiation, and migration (41, 42). The SFK-associated subfamily (BRK, FRK, and SRM) lacks C-terminal regulatory tyrosine and N-terminal myristoylation sites and is highly expressed in gastric and breast cancer (43, 44). The major activation of SFKs is often led by the highly conserved autophosphorylation site, tyrosine 416 (45), which serve as an indicator of the SFKs’ activities in the following experiments. To investigate whether SFKs regulate DNAJB6b Y53 phosphorylation, SH-SY5Y cells were incubated with 500 nM dasatinib, a pan-inhibitor of SFKs (46, 47) (Fig. 3, *D*–*F*). Under the suppression of Src family kinase activities (Fig. 3*F*), we observed a decrease in the p-Y53 level only in the V5-DNAJB6b WT group but not in the V5-DNAJB6b Y53F mutant (Fig. 3*E*). This suggests that SFKs may regulate the phosphorylation of DNAJB6b at Y53. An additional SFK inhibitor, pyrazolopyrimidine 2 (PP2), which strongly inhibits the kinase activity of FYN, LCK, and HCK (48, 49), was also tested in SH-SY5Y cells (see Fig. S4, *A–C*). Interestingly, after assuring the suppression of kinase activity (see Fig. S4*C*), the results showed no significant difference in p-Y53 level in the V5-DNAJB6b WT group (see Fig. S4*B*). Together, we predicted that the upstream kinase(s) to phosphorylate DNAJB6b at Y53 may include SRC, LYN, and YES kinase, instead of FYN, LCK, and HCK kinase, in SH-SY5Y cells.

### YES kinase directly promotes the phosphorylation of DNAJB6b Y53

To better understand the distribution of individual SFKs in human tissue, particularly in the brain, the gene expression levels were analyzed. The gene expression data were sourced from the Genotype Tissue Expression (GTEx) portal databank (https://www.gtexportal.org/home/, updated as of April 2, 2025) and the BrainSpan project (https://www.brainspan.org/static/home, updated as of April 2, 2025), respectively. In the GTEx portal databank, 54 non-diseased tissues from 19,788 samples were examined, revealing that SRC, YES, and FYN are widely expressed across tissues, whereas the others exhibit more specific distributions (see Fig. S5*A*). Additionally, five brain regions related to tau deposition (50, 51), including the medial prefrontal cortex, the superior temporal cortex, the inferolateral temporal cortex, the HIP, and the inferior parietal cortex, were selected to further assess in seven non-diseased individuals (see Fig. S5, *B* and *C*). The findings indicated that SRC, YES, FYN, and LYN (highlighted in red) exhibit higher gene expression levels than other SFKs in these brain regions (see Fig. S5*B*). Several studies have demonstrated that these SFKs may redundantly regulate neuronal survival (52), microglial migration (53), and glial phagocytic activity (54). In Fig. S5*D*, we rearranged the gene expression levels from the heatmap into a line plot. Each dot represents the z-score of gene expression in a specific brain region of the individual. The green line shows the gene expression level of FYN kinase, which remains constant across all ages. Interestingly, the gene expression levels of YES and LYN kinases follow a similar pattern, with a gradual increase before age 11 and a sudden drop at age 30. In contrast, the red line represents the gene expression level of the SRC kinase, which shows a pattern slightly opposite to that of the YES and LYN kinases. To conclude, SRC, YES, and LYN kinases may act as complementary players in signaling pathways at different ages.

**Figure 4:**
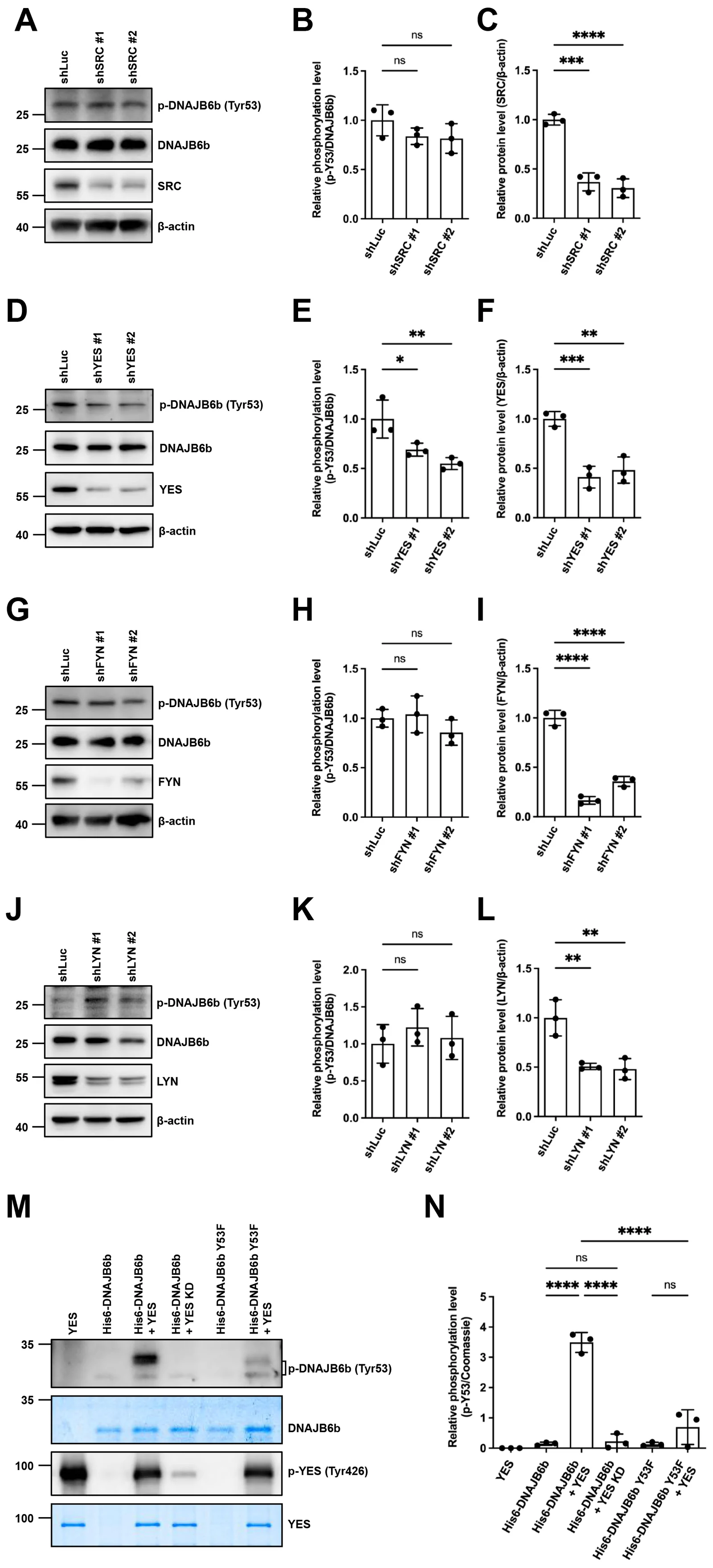
**YES, but not SRC, FYN, or LYN, phosphorylates DNAJB6b Y53 in SH-SY5Y cells and in an *in vitro* kinase assay.***A*–*L*, validation of SFKs responsible for the phosphorylation of DNAJB6b Y53. Knockdown of individual SFKs in SH-SY5Y cells was performed, including SRC (*A* and *C*, *n* = 3), FYN (*D –F*, *n* = 3), LYN (*G*–*I*, *n* = 3), and YES (*J*–*L*, *n* = 3), and analyzed by western blotting. *A*, *D*, *G*–*J*, immunoblotting with the indicated antibodies was used to assess DNAJB6b phosphorylation levels and the efficiency of SFK knockdown. β-actin served as a loading control. *B*, *E*, *H*, and *K*, quantification of the ratio of p-DNAJB6b Y53 to endogenous DNAJB6b in shSRC (*B*), shFYN (*E*), shLYN (*H*), or shYES (*K*) groups in (*A*, *D*, *G*–*J*), respectively. *C*, *F*, *I*–*L*, Quantification of the ratio of SRC (*C*), FYN (*F*), LYN (*I*), or YES (*L*) to β-actin in (*A*, *D*, *G*–*J*). *M*, assessment of YES-mediated phosphorylation at Y53 on His6-DNAJB6b WT by *in vitro* YES kinase assay. Immunoblotting with anti-p-DNAJB6b Y53 antibody and anti-p-SFK Tyr416 antibody assessed the phosphorylation level of His6-DNAJB6b and the activity of YES kinase, respectively. Coomassie *blue* staining confirmed that recombinant His6-DNAJB6b and YES kinase were equally loaded quantification of the ratio of p-DNAJB6b Y53 to Coomassie *blue*-stained His6-DNAJB6b in (*M*) (*n* = *3*). Data in (*B* and *C*, *N*, *E* and *F*, *H* and *I*, *K* and *L*, *N*) were expressed as mean ± SD, and *p*-values were calculated *via* one-way ANOVA followed by Dunnett's test. (ns, not significant; ∗*p* < 0.05, ∗∗*p* < 0.01, ∗∗∗*p* < 0.001, ∗∗∗∗*p* < 0.0001).

To verify the specific SFKs responsible for DNAJB6b Y53 phosphorylation, RNAi knockdown was performed in SH-SY5Y cells by using shSRC, shYES, shFYN, and shLYN expression plasmids. Knockdown efficiencies of SFKs were examined by Western blotting (Fig. 4, *A*–*L*). Compared to the knockdown of the firefly luciferase (Luc) control, only the knockdown of YES kinase (Fig. 4*D*), but not SRC (Fig. 4*A*), FYN (Fig. 4*G*), or LYN (Fig. 4*J*), led to a decrease in p-Y53 level. Knockdown of the four SFKs was also performed in other neuroblastoma cell lines, BE(2)-M17 cells (see Fig. S6, *A–L*). Compared to the knockdown of Luc control, a significant reduction of p-Y53 level was observed in the knockdown of YES groups (Fig. S6, *D* and *F*). These findings indicate that YES kinase plays a major role in regulating the phosphorylation of DNAJB6b Y53 in neuroblastoma cells.

To determine whether YES kinase can directly phosphorylate DNAJB6b at the Y53 site, further investigation is conducted through an *in vitro* YES kinase assay (Fig. 4, *M* and *N*). Recombinant full-length His6-DNAJB6b WT and His6-DNAJB6b Y53F proteins were overexpressed in *Escherichia coli*, purified, and used as substrates along with active YES kinase in the kinase assay (55) (Fig. 4*M*). The observed p-Y53 band is higher than expected, likely due to a molecular weight shift caused by YES kinase phosphorylating other sites on DNAJB6b. The results showed a significant increase in p-Y53 level on His6-DNAJB6b WT incubated with active YES kinase, but not with kinase-dead (KD) YES (Fig. 4*N*). Additionally, the His6-DNAJB6b Y53F mutant failed to be phosphorylated by active YES kinase. Therefore, the *in vitro* YES kinase assay demonstrates that Y53 on DNAJB6b is a direct target of YES kinase.

**Figure 5:**
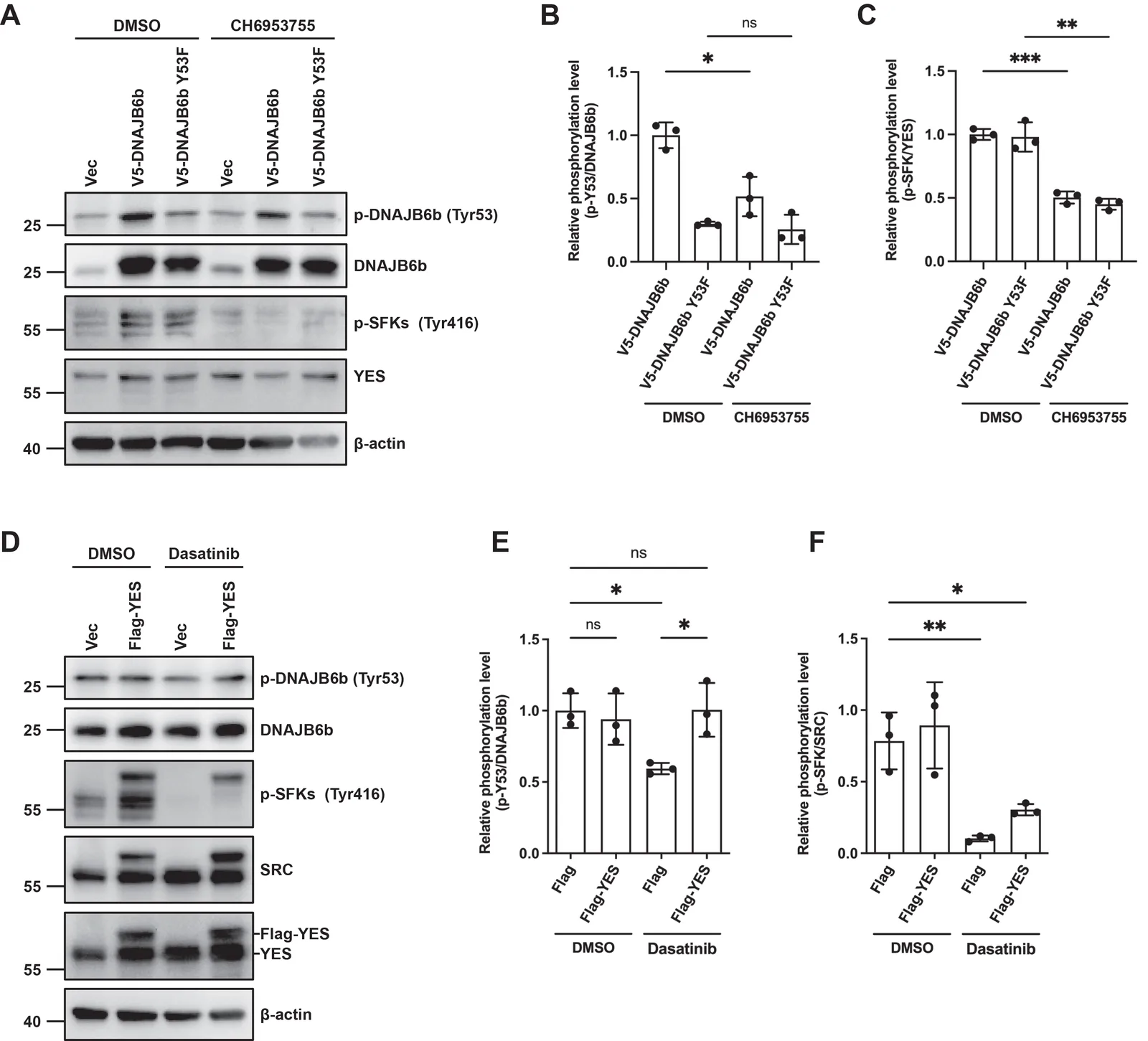
**YES specifically regulates the phosphorylation of DNAJB6b at Y53.***A*, phosphorylation of DNAJB6b at the Y53 site was evaluated in CH6953755-treated SH-SY5Y cells by western blotting. Cells were over-expressed with empty vector (pcDNA/FRT/TO-V5) and indicated V5-DNAJB6b constructs for 48 h, followed by CH6953755 treatment or solvent control DMSO for 2 h. Proteins were analyzed by immunoblotting using the indicated antibodies. *B* and *C*, quantification of the ratio of p-DNAJB6b Y53 to DNAJB6b WT or DNAJB6b Y53F (*B*) and the ratio of p-SFKs Tyr416 to YES (*C*) in (*A*) (*n* = 3). *D*, phosphorylation level of DNAJB6b Y53 was evaluated in Flag-YES-overexpressing cells after dasatinib treatment. SH-SY5Y cells were overexpressed with empty vector (pFLAG-CMV-2) or FLAG-YES for 48 h, followed by dasatinib treatment or DMSO solvent control for 2 h. Proteins were analyzed by immunoblotting using the indicated antibodies. *E and F*, quantification of the ratio of p-DNAJB6b Y53 to DNAJB6b WT (*E*) and the ratio of p-SFKs Tyr416 to SRC (*F*) in (*D*) (*n* = 3). Data in (*B* and *C*, *E* and *F*) were expressed as mean ± SD, and *p*-values were calculated *via* unpaired two-tailed Student’s *t* test for (*B* and *C*) and one-way ANOVA followed by Dunnett's test for (*E* and *F*). (ns, not significant; ∗*p* < 0.05, ∗∗*p* < 0.01, ∗∗∗*p* < 0.001).

To verify that YES kinase is responsible for the phosphorylation of DNAJB6b Y53, a selective YES inhibitor, CH6953755, is applied to confirm specificity (Fig. 5, *A*–*C*). SH-SY5Y cells were incubated for 2 h with 10 μM CH6953755. Under the suppression of kinase activity (Fig. 5*C*), a decrease in the p-Y53 level was observed only in the V5-DNAJB6b WT group but not in the V5-DNAJB6b Y53F mutant (Fig. 5*B*). Additionally, in Figure 5, *D*–*F*, we observed a slight rescue of Y53 phosphorylation following Flag-YES1 overexpression in SH-SY5Y cells after 2 h of treatment with 500 nM dasatinib. No significant difference between the level of the control group without treatment and the Flag-YES1-overexpressed group treated with dasatinib. The phosphorylation level of SFKs serves as an indicator of dasatinib treatment and of Flag-YES kinase activity. In conclusion, YES kinase is responsible for the phosphorylation of DNAJB6b at the Y53 site.

**Figure 6:**
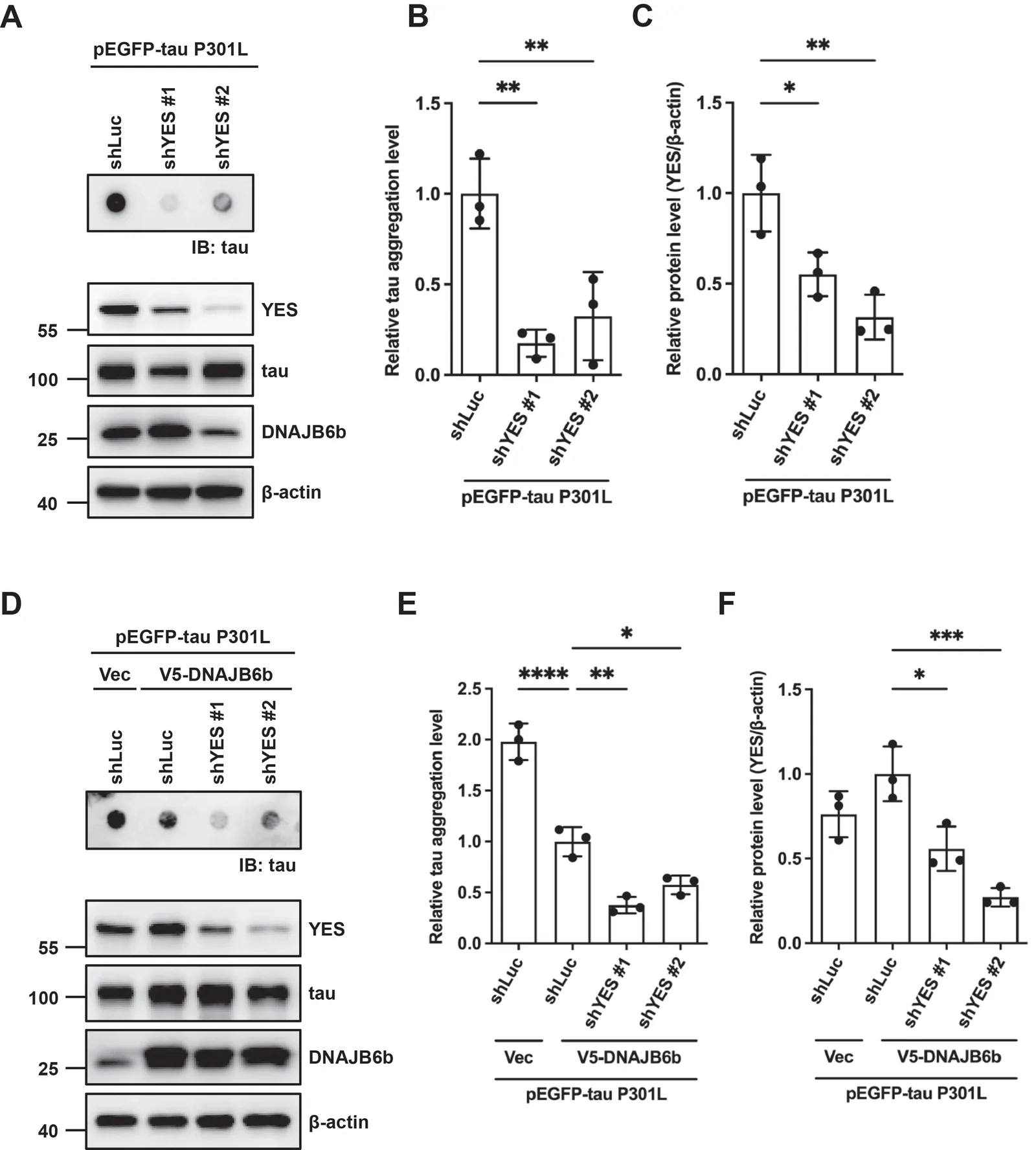
**Knockdown of YES facilitates the chaperone function of DNAJB6b to prevent tau aggregation in SH-SY5Y cells.** The filter trap assay for analysis and quantification of aggregated tau proteins after knockdown of YES. *A*, after knockdown of YES, SH-SY5Y cells were co-transfected 24 h later with EGFP-tau P301L. Retained proteins on the cellulose acetate membrane were immunoblotted using an anti-tau antibody to detect trapped tau. Immunoblotting with the indicated antibodies assessed the expression level of EGFP-tau P301L, V5-DNAJB6b, and YES. *B*, quantification of aggregated tau proteins. β-actin served as a loading control for normalization in (*A*) (*n* = 3). *C*, quantification of the ratio of YES to β-actin in (*A*). *D*, after knockdown of YES, SH-SY5Y cells were co-transfected 24 h later with EGFP-tau P301L and either the empty vector (pcDNA/FRT/TO-V5) or V5-DNAJB6b WT. Retained proteins on the cellulose acetate membrane were immunoblotted using an anti-tau antibody to detect trapped tau. Immunoblotting with the indicated antibodies assessed the expression level of EGFP-tau P301L, V5-DNAJB6b, and YES. *E*, quantification of aggregated tau proteins. β-actin served as a loading control for normalization in (*D*) (*n* = 3). *F*, quantification of the ratio of YES to β-actin in (*A*). *B* and *C*, *E* and *F*, data were expressed as mean ± SD, and *p*-values were calculated *via* one-way ANOVA followed by Dunnett's test. (∗*p* < 0.05, ∗∗*p* < 0.01, ∗∗∗*p* < 0.001, ∗∗∗∗*p* < 0.0001).

Next, we asked whether knockdown of YES kinase could restore the co-chaperone function of DNAJB6b and whether it was associated with a reduction in tau aggregates. Tau aggregation levels were assessed in YES knockdown cells, followed by overexpression of EGFP-tau P301L (Fig. 6*A*). The results showed that tau aggregation was significantly lower in YES-knockdown cells than in the shLuc group (Fig. 6, *B* and *C*). Furthermore, with the overexpression of V5-DNAJB6b WT (Fig. 6*D*), a significantly lower level of tau aggregation persisted in the YES knockdown groups (Fig. 6, *E* and *F*). The results suggest that, in the absence of the upstream kinase YES, DNAJB6b maximizes its co-chaperone activity and is associated with the decrease of tau aggregates accumulated in SH-SY5Y cells.

### Phosphorylation at Y53 on DNAJB6b increases the interaction with cytoplasmic Hsp70

**Figure 7:**
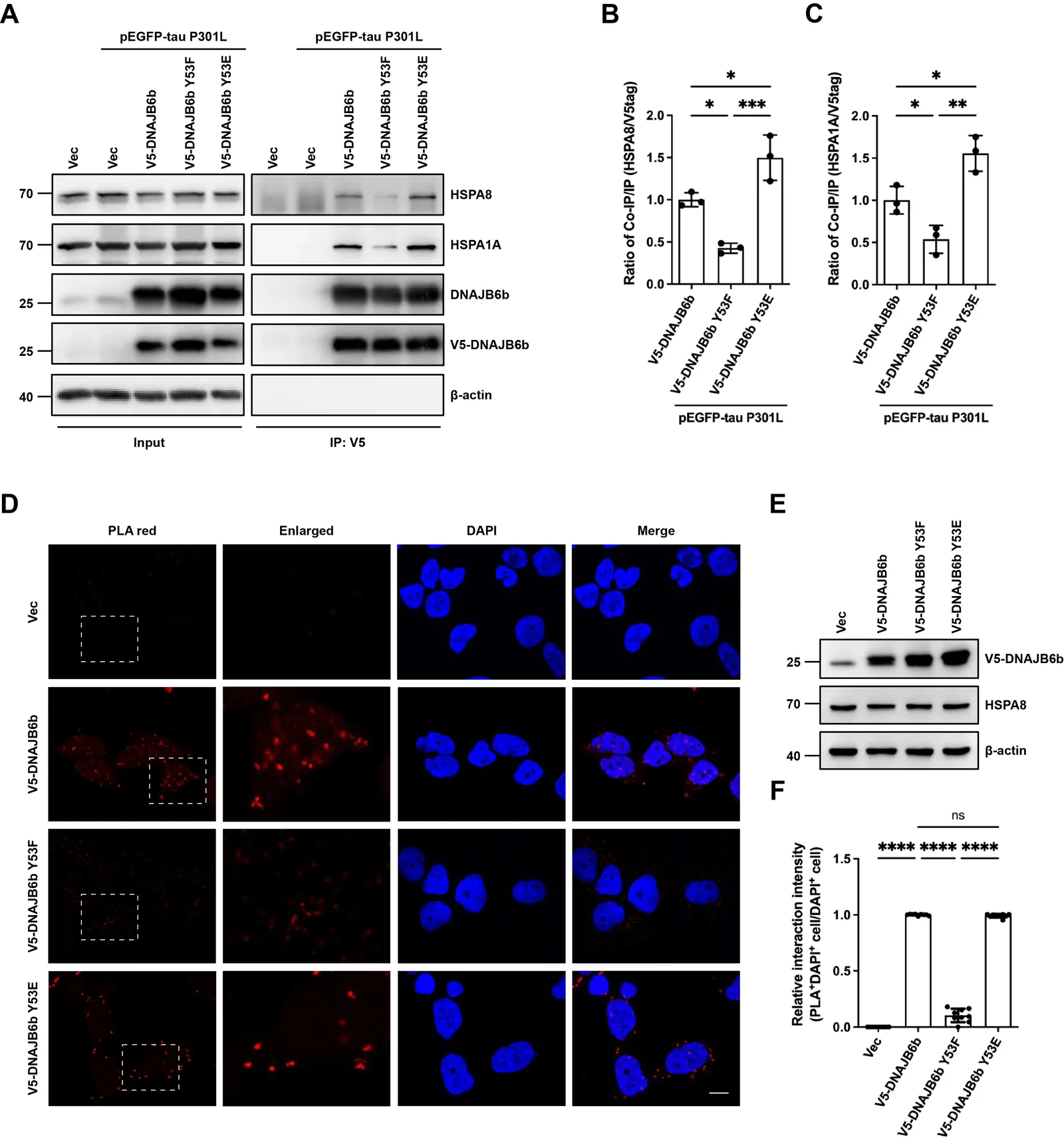
**Phosphorylation of DNAJB6b Y53 strengthens the interaction between DNAJB6b and Hsp70.***A*, The coimmunoprecipitation assay indicated the interaction between different DNAJB6b Y53 constructs and HSPA8 or HSPA1A. SH-SY5Y cells were co-expressed for 48 h with EGFP-tau P301L and the empty vector (pcDNA/FRT/TO-V5), V5-DNAJB6b WT, V5-DNAJB6b Y53F, or V5-DNAJB6b Y53E. Cell lysates were immunoprecipitated using an anti-V5 antibody to pull down V5-tagged DNAJB6b. 10% of the cell lysate from each sample was isolated to indicate the input. Precipitated and co-precipitated proteins were analyzed by immunoblotting using the indicated antibodies. *B* and *C*, quantification of the ratio of co-immunoprecipitated HSPA8 (*B*), HSPA1A (*C*) to immunoprecipitated V5-DNAJB6b WT, V5-DNAJB6b Y53F, or V5-DNAJB6b Y53E in (*A*) (*n* = 3). *D*, representative fluorescent images show the interaction between different V5-DNAJB6b constructs and HSPA8. Either the empty vector (pcDNA/FRT/TO-V5), V5-DNAJB6b WT, Y53F, or Y53E was transfected into SH-SY5Y cells. anti-V5 and anti-HSPA8 primary antibodies were hybridized to locate DNAJB6b and HSPA8, respectively. When V5-DNAJB6b and HSPA8 interact, the PLA probes on the secondary antibodies are ligated and amplified. The *red* PLA signals showing protein interaction were detected by fluorescent confocal microscopy. Nuclei were counterstained with DAPI. The scale bar represents 10 μm. *E*, immunoblotting with anti-V5 and anti-HSPA8 antibodies indicated the expression levels of different V5-DNAJB6b constructs and HSPA8, respectively. *F*, quantification of interaction intensity in (*D*) (*n* = 3). The percentage of fluorescent (PLA^+^) SH-SY5Y cells to the total number of cells (DAPI^+^) was quantified in at least 3 random-field images. Data in (*B* and *C*, *F*) were expressed as mean ± SD, and *p*-values were calculated *via* one-way ANOVA followed by Dunnett's test. (ns, not significant; ∗*p* < 0.05, ∗∗*p* < 0.01, ∗∗∗*p* < 0.001, ∗∗∗∗*p* < 0.0001). PLA, proximity ligation assay.

Previous studies indicate that the J domain of DNAJB6b interacts with HSPA8 and HSPA1A and can improve client binding, thereby helping to resolve aggregates. (21). However, it remains unclear whether DNAJB6b Y53 phosphorylation can disrupt the Hsp70 machinery's reaction cycle. To determine if DNAJB6b Y53 phosphorylation regulates the association with HSPA8 and HSPA1A in SH-SY5Y cells, a co-immunoprecipitation assay was performed (Fig. 7*A*). Compared to the V5-DNAJB6b WT group, the V5-DNAJB6b Y53F mutant showed a significant decrease in interaction with Hsp70s (Fig. 7, *B* and *C*). Meanwhile, the V5-DNAJB6b Y53E mutant exhibited a higher binding affinity than both the WT and Y53F groups, suggesting that phosphorylation at Y53 enhances the interaction between DNAJB6b and Hsp70s. To further examine the *in situ* protein-protein interactions between different DNAJB6b mutants and HSPA8, a proximity ligation assay (PLA) (56, 57) was performed (Fig. 7, *D*–*F*). Consistent with the results of the co-immunoprecipitation assay, overexpression of V5-DNAJB6b WT or Y53E showed a large number of PLA-positive puncta, indicating a strong interaction with HSPA8 after phosphorylation at Y53 (Fig. 7*F*). Overexpression of V5-DNAJB6b Y53F displayed a relatively low PLA-positive signal, suggesting that the interaction between non-phosphorylatable DNAJB6b and HSPA8 is weak. Together, the *in vitro* cell culture and *in situ* PLA data imply that Y53-phosphorylated DNAJB6b forms a strong interaction with HSPA8.

**Figure 8:**
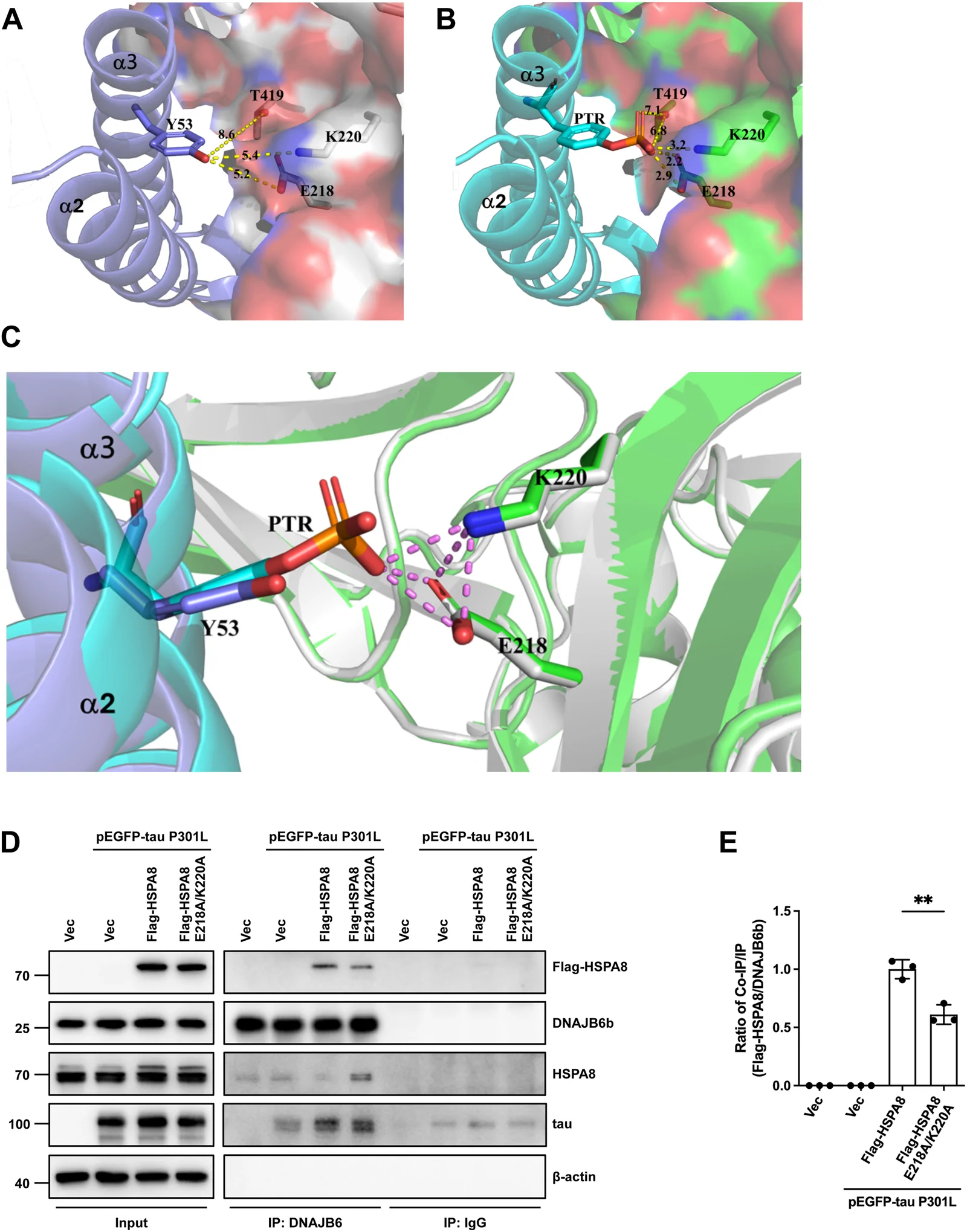
**Comparison of AlphaFold 3-predicted structures of the HSPA8-DNAJB6b complex before and after DNAJB6b Y53 phosphorylation.***A*, predicted structure of the HSPA8-DNAJB6b dimerization interface in the unphosphorylated state. DNAJB6b is shown as *purple ribbons* with Y53 in stick representation. HSPA8 is displayed in surface view using *gray*-based CPK coloring. Residues within 12.0 Å of Y53 (E218, K220, and T419) are highlighted, with *yellow dashed lines* indicating their shortest distances to Y53 (measured in Å). *B*, predicted structure of the dimerization interface upon DNAJB6b Y53 phosphorylation. DNAJB6b is shown as cyan ribbons with phosphorylated Y53 (pY53, PTR) in stick representation. HSPA8 is rendered in surface view using *green*-based CPK coloring. Residues within 12.0 Å of pY53 (E218, K220, and T419) are highlighted, and *yellow dashed lines* indicate their shortest distances to pY53 (in Å). *C*, superimposed structures of the HSPA8-DNAJB6b complex before and after Y53 phosphorylation. The phosphate group of pY53 forms a hydrogen bond network (*violet dashed lines*) with K220 and E218 of HSPA8. Coloring follows the scheme in panels (*A*) and (*B*). *D*, the coimmunoprecipitation assay indicated the interaction between Flag-HSPA8 E218A/K220A and DNAJB6b. SH-SY5Y cells were co-expressed for 48 h with EGFP-tau P301L and the empty vector (pFLAG-CMV-2), Flag-HSPA8 WT, or Flag-HSPA8 E218A/K220A. Cell lysates were immunoprecipitated with an anti-DNAJB6 antibody to pull down endogenous DNAJB6b. 10% of the cell lysate from each sample was isolated to indicate the input. Precipitated and coprecipitated proteins were analyzed by immunoblotting using the indicated antibodies. *E*, quantification of the ratio of co-immunoprecipitated Flag-HSPA8 to immunoprecipitated endogenous DNAJB6b in (*D*) (*n* = 3). Data in (*E*) were expressed as mean ± SD, and *p*-values were calculated *via* unpaired two-tailed Student’s *t* test. (ns, not significant; ∗∗*p* < 0.01).

To investigate how phosphorylation of DNAJB6b at Y53 may modulate its interaction with HSPA8, we employed AlphaFold three to predict the structure of the HSPA8-DNAJB6b complex in both unphosphorylated and phosphorylated states (Fig. 8). The modeling revealed that Y53 lies within the primary dimerization interface, with the surrounding structural features predicted at high confidence (pLDDT > 90) (See Fig. S7). In the unphosphorylated model, Y53 does not contact HSPA8 (Fig. 8*A*); however, upon phosphorylation, phosphor-Y53 (pY53) forms a plausible tripartite interaction with HSPA8 residues E218 and K220 (Fig. 8*B*), consistent with known pTyr-Lys-Glu hydrogen-bonding geometries. This rearrangement, together with the predicted phosphorylation-induced shift of the α2-α3 helices toward HSPA8, could help stabilize the complex (Fig. 8*C*). To verify the existence of the tripartite interaction between phosphor-DNAJB6b Y53 and HSPA8 residues E218 and K220, the E218A/K220A variant of human HSPA8 was generated by substituting both native GAG and AAG codons with GCG. In Figure 8*D*, the co-immunoprecipitation of endogenous DNAJB6 demonstrated that DNAJB6b significantly decreases its interaction with Flag-HSPA8 E218A/K220A. The result is consistent with structure prediction, which shows enhanced HSPA8 binding through residues E218 and K220 upon DNAJB6b Y53 phosphorylation.

## Discussion

In neurodegenerative diseases, loss of proteostasis is widely recognized as the primary hallmark of aging, leading to the accumulation of misfolded, hyperphosphorylated, or highly modified protein aggregates (2). The Hsp70 chaperone system, with the assistance of JDPs, plays a crucial role in regulating the proteostasis network. In our previous findings, DNAJB6b from the DNAJB subfamily exhibits high-affinity binding to HSPA8 and facilitates the untangling of tau aggregates (21). However, it remains unclear what role PTM plays in mediating the co-chaperone functions of DNAJB6b and its potential association with the pathogenesis of AD.

In this study, DNAJB6b Y53 phosphorylation is associated with a reduced resolution of tau aggregates. The upstream mediator, YES kinase, is then determined through the inhibitor treatment and genetic knockdown assay performed in neuroblastoma cells. Moreover, Y53 phosphorylation can be restored by YES overexpression after YES inhibitor treatment; however, the genetic knockdown of the YES group showed no differences under YES overexpression (data not shown). These results suggest that the DNAJB6b Y53 phosphorylation site might be regulated by a multifactorial signaling network. We hypothesize that the additional plasmid transfection step extends the experiment period, potentially causing an unknown effect that induces a backup pathway in response to the loss of YES kinase. While YES kinase is a primary kinase, the loss of this kinase and the transfection of an additional plasmid likely trigger compensatory intervention from other unknown cellular factors/kinases/pathways.

Using the immunoprecipitation assay, we observed an increase in *in situ* interaction between DNAJB6b Y53E and Hsp70s, particularly HSPA8 and HSPA1A. Y53 phosphorylation may influence the autoinhibitory helix V, which has been reported to restrict access of the His-Pro-Asp motif to Hsp70 (23). The predicted conformational changes near the α2-α3 region could allosterically perturb helix V positioning, thereby relieving this auto-inhibition and facilitating HSPA8 engagement. Based on previous studies, the LGMDD1 mutations cause DNAJB6b to undergo a "toxic gain-of-function". The co-chaperone becomes unregulated, binding Hsp70 too tightly and preventing the machinery from releasing the folded protein (23). In our study, the phosphorylation status of DNAJB6b may have a similar impact to mutations in DNAJB6 that cause LGMDD1, whereby YES-mediated phosphorylation of DNAJB6b Y53 may interfere with the auto-inhibition of helix V, and thereby facilitating Hsp70 engagement. When phosphorylated, DNAJB6b′s function may remain intact; however, it might sequester Hsp70s away from other proteins that are essential for clearing aggregated tau and/or for the next round of the folding cycle.

Aging is a significant risk factor for AD, potentially due to cellular and molecular changes associated with advancing age (58). One contributing mechanism may involve increased activation of receptor tyrosine kinases and the SFK pathways during aging. These pathways are critical for promoting cellular proliferation and survival, which are advantageous for aged cells and tissues (58, 59, 60). While higher Y53 phosphorylation is detected in the brain lysate of the AD patient, its effect on DNAJB6b′s function and linkage to AD cannot be assessed in this state. However, we hypothesize that the enhanced activity of receptor tyrosine kinases and SFKs comes at a cost: the downregulation of the DNAJB6b-mediated Hsp70 folding pathway, an essential component of proteostasis. This compromise in proteostasis increases the likelihood of tau protein aggregation and Aβ plaque formation, hallmark features of AD pathology. Consequently, while aged cells benefit from improved survival mechanisms, they become more susceptible to the molecular dysfunctions underlying AD progression.

## Conclusions

Overall, this study shows that dephosphorylation of Y53 in DNAJB6b is associated with reduced tau aggregates. The links between Y53-phosphorylated DNAJB6b and the levels of pathological phospho-tau in AD brains support our conclusion that Y53-phosphorylated DNAJB6b may disrupt the folding cycle because of the excessive interaction with chaperones.

## Experimental procedures

### Cell lines and culture conditions

SH-SY5Y and BE(2)-M17 neuroblastoma cells were obtained from the American Type Culture Collection and cultured in Dulbecco's Modified Eagle Medium/Nutrient Mixture F-12 (DMEM/F12) medium (Cytiva) supplied with 10% fetal bovine serum (VWR) and 1x penicillin/streptomycin/amphotericin B (Capricorn Scientific). Cells were plated on poly-L-lysine-coated 10 cm cell culture dishes or 6-well culture plates and grown in a humidified incubator at 37 °C with 5% CO_2_.

### Plasmids

Primers used for plasmid generation and the specific oligonucleotide sequences of the shRNAs are listed in Document S1: Tables S4 and S5. Construction of pEGFP-C1-tau P301L (2N4R) and pcDNA5/FRT/TO-V5 control was performed as described (21), while pFLAG-CMV-2 YES1 and pFLAG-CMV-2-hHSPA8 E218A/K220A were prepared with the help of the Biomedical Resource Core at the First Core Labs, National Taiwan University College of Medicine. The VN-tau (P301L), tau (P301L)-VC, and pcDNA5/FRT/TO-V5-DNAJB6b plasmids were purchased from Addgene (Watertown).

The pcDNA5/FRT/TO-V5-DNAJB6b Y53F and pcDNA5/FRT/TO-V5-DNAJB6b Y53E plasmids were generated by site-directed mutagenesis using HiFi polymerase (Kapa Biosystems) with DNAJB6-Y53F-BstBI-For and DNAJB6-Y53E-NdeIdel-For, respectively. Meanwhile, the E218A/K220A variant of hHSPA8 was generated in the pFLAG-CMV-2 backbone by substituting both the native GAG and AAG codons with GCG. All plasmids were sequenced before use and are listed in Document S1: Table S6.

### Transient transfections, shRNA transfections, and sample preparation

Either transient transfections or shRNA transfections were performed using Lipofectamine LTX reagent with PLUS reagent (Thermo Fisher Scientific) according to the manufacturer’s instructions. Cells were grown to 60 to 70% confluence on poly-D-lysine-coated 6-well plates and transfected for 48 to 72 h. For shRNA transfection, cells were additionally selected with 2 to 4 μg/ml of puromycin (Sigma-Aldrich) for at least 2 days. Cell pellets were collected and lysed on ice for 10 min in RIPA lysis buffer (Merck Millipore), then centrifuged at 13,000 rpm for 15 min at 4 °C. The supernatants were collected, and the protein concentration was determined by the Bio-Rad Protein Assay (Bio-Rad Laboratories). 5x SDS sample buffer (250 mM Tris-HCl, pH 6.8, 10% SDS, 0.25% bromophenol blue, 50% glycerol, 10 mM DTT, 0.5 M 2-mercaptoethanol) was then added to the supernatants, and the samples were heated at 110 °C for 15 min. Samples were stored at −80 °C for further analysis.

### Filter trap assay

The filter trap assay was performed as previously described (21, 61). For the DNAJB6 wild-type and tau P301L groups, cells were co-transfected with 1.5 μg and 0.75 μg of plasmids, respectively. For the DNAJB6b mutant and tau P301L groups, 1.5 μg and 0.5 μg of plasmid were used, respectively. Cell pellets were collected and lysed with 1% Triton X-100 in PBS solution supplemented with 1 mM PMSF, 1x cOmplete EDTA-free Protease Inhibitor Cocktail (Roche), and 1x PhosSTOP Phosphatase Inhibitors (Roche). After a total of 20 s of sonication, the protein concentration was determined using Bio-Rad Protein Assay (Bio-Rad Laboratories) and diluted to a final concentration of 1 μg/μl with lysis buffer containing 1% SDS. 60 to 90 μl solution per sample was then passed through 0.2 μm cellulose acetate membranes (Sterlitech) using a 96-well dot blot apparatus (Bio-Rad Laboratories). Before filtration, the membranes were prewashed with 0.1% SDS in PBS solution. Proteins trapped by the membranes were detected by immunostaining following the protocols described in immunoblotting.

### Immunoblotting

A total of 15 to 30 μg protein was resolved by SDS-polyacrylamide gel electrophoresis and transferred to a polyvinylidene difluoride membrane (Merck Millipore). The membrane was blocked in 5% nonfat milk or 5% BSA at room temperature for 1 h, and then incubated with primary antibodies at 4 °C overnight. Primary antibodies (Document S1: Table S7) were used to detect tau (1:1000, GeneTex), GFP (1:2000, GeneTex), Phospho-DNAJB6b (Tyr53) (1:250, this study), DNAJB6 (1:2000, Abcam), V5 (1:1000, Abcam), AT8 (1:500, Thermo Fisher Scientific), Phospho-AKT (Ser473) (1:1000, Cell Signaling Technology), AKT (1:1000, Cell Signaling Technology), Phospho-Src Family (Tyr416) (1:500, Cell Signaling Technology), SRC (1:1000, Cell Signaling Technology), FYN (1:1000, GeneTex), LYN (1:1000, GeneTex), YES (1:500, Cell Signaling Technology), FLAG (1:1000, Sigma-Aldrich), HSPA8 (1:1000, Novus Biologicals), HSPA1A (1:1000, Santa Cruz Biotechnology), and β-actin (1:2000, Proteintech). Next, the membrane was washed 3 times with 1x Tris-buffered saline with Tween-20 (20 mM Tris-HCl, pH 7.6, 150 mM NaCl, 0.1% Tween-20), followed by incubation with the horseradish peroxidase-conjugated goat anti-rabbit or goat anti-mouse IgGs (1:5000, Jackson ImmunoResearch) at room temperature for at least 1 h. Luminata Crescendo Western HRP Substrate (Merck Millipore) was used for visualization by ChemiDoc Imaging System (Bio-Rad Laboratories). The images were quantified by Image Lab software (Bio-Rad Laboratories; https://www.bio-rad.com/en-tw/product/image-lab-software?ID=KRE6P5E8Z).

### BiFC assay

The BiFC assay was performed as described in previous studies (21, 33). SH-SY5Y cells were seeded at 2 × 10^5^ cells/ml onto square glass coverslips (Marienfeld Laboratory Glassware) in 6-well plates. After 24 h of incubation, cells were transfected with different DNAJB6b constructs, along with plasmids that encode full-length tau P301L protein fused to the N-terminal part of Venus protein, VN-tau P301L (Addgene), and tau P301L protein fused to the C-terminal part of Venus protein, tau P301L-VC (Addgene). After 48 h of transfection, cells were fixed with 4% paraformaldehyde in 1x PBS for 15 min at room temperature, followed by permeabilization with 0.1% Triton X-100 in 1x PBS for 5 min. The coverslips were blocked with 1% BSA for 90 min, then immunostained overnight with V5-DNAJB6 (anti-V5 antibody, 1:100, Thermo Fisher Scientific). Next, the coverslips were washed 3 times with 1x PBS, followed by 1-h incubation with the Alexa Fluor 594 Goat anti-Mouse IgG (1:200, Invitrogen) at room temperature. Afterward, coverslips were washed with 1x PBS and counterstained with 4′, 6-diamidino-2-phenylindole (DAPI) (1:1000, Thermo Fisher Scientific) for 5 min. Finally, coverslips were mounted with 25 μl of Fluoromount Aqueous Mounting Medium and imaged using a Zeiss Axio Imager.M2 fluorescence microscope.

The transfection efficiency of the plasmids was verified by immunoblotting with anti-DNAJB6 (Abcam) and anti-tau (GeneTex) antibodies. To quantify the relative number of Venus-tau aggregates formed in cells, 3 random-field images were taken at 10× magnification in each condition. Cell numbers were identified through ImageJ (NIH). Captured images were inverted into 16 bit type and counted using a size threshold of >10 in^2^ and a circularity index of 0 to 1.0. At least 100 to 500 cells were analyzed per field across 3 independent biological replicates. The actual cell count in each field is listed in the Supplementary File (Excel). BiFC fluorescence levels are quantified as the number of fluorescent (BiFC+) SH-SY5Y cells divided by the total number of cells (DAPI^+^). Final representative images were taken at 10x magnification and were also included in the quantification of BiFC fluorescence levels. Images were viewed using Zen Blue 2.6 (Zeiss; https://www.zeiss.com/microscopy/en/products/software/zeiss-zen.html).

### Preparation of phosphor-DNAJB6b (Tyr53) polyclonal antibodies

Synthesized peptide with a length of 12 amino acids and an additional cysteine on the C-terminal for Keyhole Limpet Hemocyanin (KLH) conjugation: A-E-A-(pY)-E-V-L-S-D-A-K-K-C (MW: 1506.57) is ordered from Synbio technologies. KLH conjugation was performed utilizing the ImjectTM Maleimide-Activated mcKLH Spin Kit following the protocols prescribed by the manufacturer (Thermo Fisher Scientific). 50 μl of the peptide-KLH conjugates was administered intraperitoneally into a naïve BALB/c mouse. After 3 weeks, the previously primed mouse received two booster injections of the peptide-KLH. Animal experiments were performed in accordance with guidelines and approved by the Institutional Animal Care and Use Committee at the Laboratory Animal Center, College of Medicine, National Taiwan University.

The affinity and specificity of phosphor-DNAJB6b Tyr53 polyclonal antibodies were identified through Western blotting and filter trap assay. For the latter, 0.05, 0.5, and 5 μg of the phosphorylated or unphosphorylated peptides were spotted onto 0.2-μm nitrocellulose membranes (Bio-Rad Laboratories). Filter trap assay analysis was conducted by standard protocol using phosphor-DNAJB6b Y53 antibody.

### Human brain lysate samples

Non-AD control (NB820-59177, GTX27918, GTX27918) and AD (NB820-59363, GTX26622) post-mortem whole brain lysates were purchased from Novus Biologicals and Genetex. For biological replicates, products from different individuals were used. Product #NB820-59177 includes non-AD brain lysates from a 26-year-old Asian male (lot #C511134), an 82-year-old Caucasian male (lot #C807591), and a 71-year-old Caucasian male (lot #C910514), all of whom had no clinical evidence of neurological disorders. Product #GTX28771 was obtained from an 82-year-old Caucasian male (lot #822400674), who had known pathologies of aortic stenosis. Product #GTX27918 was obtained from a 71-year-old Caucasian male (lot #822600376) with no clinical evidence of neurological disorders. Products #NB820-59363 and #GTX26622 contain brain lysates from individuals with confirmed AD pathology, including a 93-year-old Hispanic female (lot #C511136), an 87-year-old Caucasian male (lot #C511137), a 93-year-old Hispanic female (lot #C910505), a 93-year-old female (lot #822304853), and an 87-year-old female (lot #822502057). All individual details are listed in Document S1: Table S8.

### Preparation of stock solutions and treatment conditions

NVP-AEW541, dasatinib, PP2, and CH6953755 were purchased from MedChemExpress (Monmouth Junction) for inhibitor treatments. All inhibitors were dissolved in DMSO (Sigma-Aldrich) to a final concentration of 10 mM as a stock solution.

To determine the final treatment dosage, 0.01 to 100 μM of NVP-AEW541, dasatinib, and CH6953755 were applied to SH-SY5Y cells for 2 h (37, 46, 62); PP2 was applied for 6 h (48, 63). The IC50 values for NVP-AEW541, dasatinib, PP2, and CH6953755 were calculated to be 0.3207, 0.0603, 1.155, and 0.5768, respectively (Fig. S3). The final concentrations of each inhibitor were then determined to be 10 μM, 500 nM, 10 μM, and 1 μM.

For NVP-AEW541, the activity of the insulin receptor kinase was determined through the phosphorylation of AKT on serine 473. For dasatinib, PP2, and CH6953755, SFKs’ activity was determined through the phosphorylation of SFKs on tyrosine 416.

### Expression analysis

The tissue gene expression data were obtained from the GTEx portal database (https://www.gtexportal.org/home/) on April 2nd, 2025. The GTEx Multi-Gene Query platform was used to investigate gene expression levels of 11 SFKs, including SRC, YES1, FYN, FGR, LYN, HCK, LCK, BLK, PTK6 (BRK), FRK, and SRMS (SRM), across 54 non-diseased tissues from 19,788 samples. The TPM (Transcripts per million) metric was used to quantify gene expression levels.

The gene expression data of 11 SFKs and DNAJB6 from five brain regions, the medial prefrontal cortex, the superior temporal cortex, the inferolateral temporal cortex, the HIP, and the inferior parietal cortex, were obtained from the BrainSpan project database (https://www.brainspan.org/static/home) on April 2nd, 2025. The log_2_ RPKM (reads per kilobase per million) values in seven individuals at different ages (12 pcw, 24 pcw, 1, 11, 21, 30, and 37 years old) were downloaded and converted into z-scores for visualization in a heatmap by the following equation:

$$z-score=\frac{\left(\log\;2\;RPKM\right)-Means\;of\;all\;analyzed\;genes}{Standard\;deviation\;of\;all\;analyzed\;genes}$$

The original search can be reproduced on the BrainSpan website at: https://www.brainspan.org/rnaseq/searches?search_type=user_selections.

### Recombinant His6-DNAJB6b purification and *in vitro* YES kinase assay

The recombinant full-length His6-DNAJB6b WT and Y53F were constructed by the T4 ligation method. pcDNA5/FRT/TO-V5-DNAJB6b WT and Y53F were digested with BamHI-HF and NotI-HF as “insert” and ligated to the restriction enzyme-digested pET-28a vector with T4 ligase. Then, the recombinant His6-DNAJB6b WT and Y53F were produced in BL21(tRNA) bacteria, followed by IPTG induction, and purified by affinity to histidine-tagged protein purification resin (Cytiva). Protein concentration was determined by the Bio-Rad Protein Assay (Bio-Rad Laboratories). The purified His6-DNAJB6b was submitted to buffer exchange by using an Amicon Ultra-0.5 Centrifugal Filter Unit (3 kDa, Merck Millipore) and stored in 1x tyrosine kinase reaction buffer (20 mM HEPES pH 7.4, 1 mM MnC1_2_, 1 mM DTT, 100 μM Na_3_VO_4_) with 20% glycerol at −80 °C.

For *in vitro* YES kinase assays, 2 nM His6-DNAJB6b or His6-DNAJB6b Y53F was incubated with 10 ng active GST-YES kinase (Thermo Fisher Scientific) and 200 μM ATP in tyrosine kinase reaction buffer for 15 min at 30 °C. The reactions were quenched by adding 7.5 μl of 5x sample buffer and then boiled at 110 °C for 10 min. Samples were analyzed by western blotting, and the homogeneity of the recombinant His6-DNAJB6b was checked by Coomassie blue staining.

### Co-immunoprecipitation assay

SH-SHY5Y cells were harvested and lysed for 30 min in immunoprecipitation buffer (50 mM Tris–HCl pH 7.5, 150 mM NaCl, 0.5% Triton X-100, 1 mM EDTA), supplemented with 1 mM PMSF, 1x Complete EDTA-free Protease Inhibitor Cocktail (Roche), and 1x PhosSTOP Phosphatase Inhibitors (Roche). 2 μg anti-V5 antibody (Abcam) or 2.5 μg anti-DNAJB6 (Abcam) was mixed with a final concentration of 2 μg/ml lysate and incubated tumbled end-over-end for 1 h at 4 °C. Lysates were then incubated with Protein G Mag Sepharose Xtra magnetic beads (Cytiva) for 90 min at 4 °C. After 3 extensive washes with wash buffer (50 mM Tris-HCl, pH 7.5, 150 mM NaCl, 0.5% Triton X-100, 1 mM EDTA), the bound proteins were eluted by adding 20 to 60 μl of 2x SDS sample buffer. Precipitates were boiled for 10 min at 110 °C before analysis by SDS-polyacrylamide gel electrophoresis.

### PLA

The PLA signal was observed as distinct fluorescent puncta, indicating proximity (<40 nm) between the targeted proteins. To determine the *in situ* interaction between different DNAJB6b constructs and HSPA8, PLA was performed using the Duolink *In Situ* Red Starter Kit Mouse/Rabbit (Sigma-Aldrich), according to the manufacturer’s protocol. SH-SY5Y cells were seeded at 1 × 10^4^ cells/ml onto circular glass coverslips (Marienfeld Laboratory Glassware) in 12-well plates and were transfected with different DNAJB6b constructs. After 48 h of transfection, cells were fixed with 4% paraformaldehyde in PBS for 15 min at room temperature, then permeabilized with 0.1% Triton X-100 in PBS for 5 min. The coverslips were then blocked with 1x Duolink blocking solution for 30 min and incubated with anti-V5 (1:50, Thermo Fisher Scientific) and anti-HSPA8 (1:250, Novus) antibodies in Duolink antibody diluent at 4 °C overnight. After incubation, coverslips were washed twice with Duolink Wash Buffer A and incubated with both PLA PLUS and PLA MINUS probes in a 1:5 dilution for 1 h at 37 °C. Ligation (1:40 in ligase solution) was carried out for 30 min, followed by amplification (1:80 in amplification solution) for 100 min using Detection Reagents Red. Afterward, coverslips were washed with Duolink Wash Buffer B, and the nuclei were stained with DAPI (1:1000, Thermo Fisher Scientific). Coverslips were later mounted with 25 μl of Duolink *in situ* mounting medium and imaged using a Zeiss LSM880 confocal microscope.

To quantify the relative interaction intensity, a Zeiss LSM880 fluorescence microscope was used. The quantification was plotted as the number of cells with a PLA signal divided by the total number of cells (PLA^+^DAPI^+^/DAPI^+^ cells). Cell numbers were identified through ImageJ (NIH). Captured images were inverted into 16-bit type and counted based on a size threshold of >10 in^2^ and a circularity index of 0 to 1.0. At least 80 to 200 cells were analyzed per condition. The actual cell count in each field is listed in the Supplementary file (Excel). Typically, over 3 random-field images were acquired at 40× magnification for each condition in each independent experiment. The total of 3 independent biological replicates was performed. The final representative images were acquired at 63× magnification. Images were viewed using Zen Blue 2.6 (Zeiss).

### Protein docking studies

The full-length amino-acid sequences of human HSPA8 (UniProt E9PS65) and DNAJB6b (UniProt O75190) were submitted to the AlphaFold three server (https://alphafoldserver.com/welcome) for multimeric complex prediction. Two DNAJB6b models were generated: one with unmodified Y53 and another with phosphorylated Y53. The resulting unphosphorylated and phosphorylated complexes were imported into the PyMOL Molecular Graphics System (Version 3.1.3.1; Schrödinger, LLC) for structural analysis and superposition. Cα atoms corresponding to residues 1 to 500 of HSPA8 were aligned using the *super* command, yielding an overall RMSD of 0.25 Å.

### Statistical analysis

The protein levels in western blotting and filter trap assays were quantified using Image Lab software (Bio-Rad Laboratories). All data are presented as mean values ± SD, calculated using Numbers version 14.3 (Apple) and presented using GraphPad Prism nine version 9.5.1 (GraphPad Software). All experiments were performed in a minimum of 3 biological replicates. The statistical test performed for each experiment was: an unpaired two-tailed Student’s *t* test or one-way ANOVA, followed by Dunnett's test for multiple comparisons. *p*-values < 0.05 were considered statistically significant and were indicated in the figures. (ns, not significant; ∗*p* < 0.05; ∗∗*p* < 0.01; ∗∗∗*p* < 0.001; ∗∗∗∗*p* < 0.0001).

### Ethics approval and consent to participate

All animal studies were performed according to the guidelines and approved by the Institutional Animal Care and Use Committee at the Laboratory Animal Center, College of Medicine, National Taiwan University.

## Data availability

All data are available upon reasonable request and directed to the corresponding authors. All materials generated in this study are available from the corresponding authors with a completed Material Transfer Agreement.

## Supporting information

This article contains supporting information. Supporting information PDF. Include Figures S1–S8 and Tables S1–S10.

Supplementary File (Excel). The individual data values of the replicates, related to Figure 1, Figure 2, Figure 3, Figure 4, Figure 5, Figure 6, Figure 7, Figure 8, S1–S6.

## Consent for publication

Not applicable.

## Conflict of interest

The authors declare that they have no conflicts of interest with the contents of this article.
