# Supplementary material for: The Src family kinase YES regulates DNAJB6b chaperone activity to modulate tau aggregation in Alzheimer’s disease
Source: J Biol Chem. 2026 Jul 24;302(9):113363. doi: 10.1016/j.jbc.2026.113363 (PMC13503143; doi:10.1016/j.jbc.2026.113363)
Supplement: Supplementary file 1 [file mmc2.docx]

**Figure S1. Validation of phosphor-specific antibody of DNAJB6b Tyr53.**

**(A)** The anti-p-DNAJB6b Y53 antibody recognized the phosphor-peptides of DNAJB6b Tyr53 in the filter trap assay. **(B)** Validation of anti-p-DNAJB6b Y53 antibody using western blotting. SH-SY5Y cells were transfected with either the empty vector (pcDNA/FRT/TO-V5), V5-DNAJB6b wild-type, Y53F, or Y53E. The expression of different V5-DNAJB6b constructs was assessed by immunoblotting using an anti-DNAJB6 antibody. β-actin served as a loading control. **(C)** Quantification of the ratio of p-DNAJB6b Y53 to DNAJB6b wild-type, Y53F, or Y53E in **(B)** (*n*=3). Data were represented as mean ± SD, and *p*-values were calculated via one-way ANOVA followed by Dunnett´s test. (*** *p*  <  0.001, **** *p*  <  0.0001)

**Figure S2. Overexpression of EGFP-tau P301L downregulates the phosphorylation of DNAJB6b Y53 in SH-SY5Y cells.**

**(A)** The phosphorylation of DNAJB6b at the Y53 site was evaluated in SH-SY5Y cells by western blotting. SH-SY5Y cells were transfected with either the empty vector (pEGFP-C1), EGFP-tau P301L, or wild type. The expression of different EGFP-tau constructs was assessed by immunoblotting with tau antibodies. β-actin served as a loading control. **(B)** Quantification of the ratio of p-DNAJB6b Y53 to DNAJB6b wild-type in **(A)** (*n*=3). Data were represented as mean ± SD, and *p*-values were calculated via one-way ANOVA followed by Dunnett´s test. (ns, not significant; * *p*  <  0.05)

**Figure S3. Dose-response curves for NVP-AEW541, Dasatinib, PP2, and CH6953755 (0.01-100 μM), with DMSO as a solvent control.**

**(A)** Inhibition of AKT S473 autophosphorylation by NVP-AEW541, a selective InsR kinase inhibitor. 0.01-100 μM of NVP-AEW541 was applied to SH-SY5Y cells for 2 hours. **(B)** Inhibition of SFK Y416 autophosphorylation by dasatinib, a pan-inhibitor of SFKs. 0.01-100 μM of dasatinib was applied to SH-SY5Y cells for 2 hours. **(C)** Inhibition of SFK Y416 autophosphorylation by PP2, a selective inhibitor of FYN, LCK, and HCK. 0.01-100 μM of PP2 was applied to SH-SY5Y cells for 6 hours. **(D)** Inhibition of SFK Y416 autophosphorylation by CH6953755, a selective inhibitor of YES. 0.01-100 μM of CH6953755 was applied to SH-SY5Y cells for 2 hours. **(E)** Dose-response curves for NVP-AEW541 (red), Dasatinib (blue), PP2 (orange), and CH6953755 (green) (0.01-100 μM). All data points are based on single tests shown in panels **(A)-(D)**. The bottom value was set to 0, and the top values were 90.25, 85.77, 84.24, and 69.57, respectively. The IC50 values were calculated as 0.3207 μM, 0.0603 μM, 1.155 μM, and 0.5768 μM, respectively.

**Figure S4. Src family kinases, other than FYN, LCK, and HCK, mediate the phosphorylation of DNAJB6b at Y53. (Related to Figure 3)**

**(A)** The phosphorylation level of DNAJB6b Y53 was evaluated in PP2-treated cells by western blotting. SH-SY5Y cells were overexpressed with empty vector (pcDNA/FRT/TO-V5) and indicated V5-DNAJB6b constructs for 48 hours, followed by 10 μM PP2 treatment or DMSO solvent control for 6 hours. Proteins were analyzed by immunoblotting using the indicated antibodies. **(B-C)** Quantification of the ratio of p-DNAJB6b Y53 to DNAJB6b wild-type or DNAJB6b Y53F **(B)** and the ratio of p-SFKs Tyr416 to SRC, YES, FYN, or LYN (**C**) in (**A**) (*n*=3). Data in (**B-C**) were expressed as mean ± SD, and *p*-values were calculated via unpaired two-tailed Student’s t-test. (ns, not significant; * *p*  <  0.05, ** *p*  <  0.01, *** *p*  <  0.001)

## Figure S5. Gene expression profiles of individual SFKs in human tissues and various brain regions.

The mRNA expression of 11 Src family kinases, including SRC, YES1, FYN, FGR, LYN, HCK, LCK, BLK, PTK6 (BRK), FRK, and SRMS (SRM), in human tissues. **(A)** The heatmap presents the mRNA expression of individual SFKs in 54 non-diseased tissues, derived from 19,788 samples. Data was downloaded from the GTEx Multi-Gene Query platform from the GTEx portal databank (<https://www.gtexportal.org/home/>, updated as of April 2, 2025). The TPM metric was used to quantify gene expression levels. **(B)** The heatmap presents the mRNA expression of individual SFKs in five brain regions from **(C)** in seven non-diseased individuals. Data were downloaded from the BrainSpan project database (<https://www.brainspan.org/static/home>) on April 2^nd^, 2025. The log_2_ RPKM values in seven individuals at different ages (12 pcw, 24 pcw, 1, 11, 21, 30, and 37 years old) were downloaded and converted into z-scores for visualization. The graph was plotted with GraphPad Prism 9 software. **(C)** Five brain regions associated with tau deposition are highlighted in different colors. The medial prefrontal cortex (MFC) is marked as blue; the superior temporal cortex (STC) as the upper side of the purple region; the inferolateral temporal cortex (ITC) as the lower side of the purple region; the hippocampus (HIP) as dark blue, and the inferior parietal cortex (IPC) as light yellow. The illustration was created with BioRender.com. **(D)** The line plots present the mRNA expression pattern of four individual SFKs in five brain regions from **(C)** in seven non-diseased individuals at different ages (12 pcw, 24 pcw, 1, 11, 21, 30, and 37 years old). Data were downloaded and processed as described in **(B)**. The gene expression pattern of SRC, YES1, FYN, and LYN is marked in red, orange, green, and blue, respectively. Each dot represents the z-score of gene expression in a specific brain region of the individual. The graph was plotted with GraphPad Prism 9 software. (pcw: post conception weeks)

## Figure S6. YES is responsible for DNAJB6b Y53 phosphorylation in BE(2)-M17 cells. (Related to Figure 4)

**(A-L)** Validation of SFKs responsible for the phosphorylation of DNAJB6b Y53. Knockdown of individual SFKs in BE(2)-M17 cells was performed, including SRC (**A-C**) (*n*=3), FYN (**D-F)** (*n*=3), LYN (**G-I)** (*n*=3), and YES (**J-L)** (*n*=3), and analyzed by western blotting. **(A, D, G, J)** Immunoblotting with the indicated antibodies was used to assess the phosphorylation level of DNAJB6b and the knockdown efficiency of SFKs. β-actin served as a loading control. **(B, E, H, K)** Quantification of the ratio of p-DNAJB6b Y53 to endogenous DNAJB6b in shSRC **(B)**, shFYN **(E)**, shLYN **(H)**, or shYES **(K)** groups in **(A, D, G, J)**, respectively. **(C, F, I, L)** Quantification of the ratio of SRC **(C)**, FYN **(F)**, LYN **(I)**, or YES **(L)** to β-actin in **(A, D, G, J)**. Data in **(B-C, E-F, H-I, K-L, N)** were expressed as mean ± SD, and *p*-values were calculated via one-way ANOVA followed by Dunnett´s test. (ns, not significant; * *p*  <  0. 05, ** *p*  <  0.01)

## Figure S7. Quality assessment of AlphaFold 3-predicted structures of the HSPA8-DNAJB6b complex before and after DNAJB6b Y53 phosphorylation. (Related to Figure 8)

**(A)** Predicted structure and confidence metrics of the HSPA8-DNAJB6b complex in the unphosphorylated state. **(B)** Predicted structure and confidence metrics of the complex following DNAJB6b Y53 phosphorylation. Both models are color-coded by the predicted local distance difference test (pLDDT) scores across the entire complex, as indicated by the accompanying gradient scale. The PAE heatmaps show the predicted aligned error (in Å) for all residue pairs, ranging from light green (higher error) to dark green (lower error). Black boxes highlight the specific region containing Y53 in **(A**) and pY53 in **(B**), both of which were predicted with high confidence within the overall high-confidence assembly.

**Figure S8. Images of the full immunoblots.**

**Table S1. The phosphorylation sites on DNAJB6 were reported in the PhosphoSitePlus database.**

| Site | Amino acid sequence of DNAJB6 | LTP | HTP |
| --- | --- | --- | --- |
| Y4 | ____MVD(pY)YEVLGVQ | 0 | 1 |
| S15 | LGVQRHA(pS)PEDIKKA | 0 | 7 |
| Y53 | FKQVAEA(pY)EVLSDAk | 0 | 14 |
| S57 | AEAYEVL(pS)DAKKRDI | 0 | 1 |
| Y65 | DAKKRDI(pY)DKYGKEG | 0 | 4 |
| S226 | EEDGQLK(pS)LTINGVA | 0 | 1 |
| T228 | DGQLKSL(pT)INGVADD | 0 | 1 |
| S267 | PKPPRPA(pS)LLRHAPH | 0 | 3 |
| S277 | RHAPHCL(pS)EEEGEQD | 0 | 24 |
| S316 | KQKQREE(pS)KKKKSTK | 0 | 1 |

Candidates of the DNAJB6b PTM site are assessed through the available data from PhosphositePlus® (<https://www.phosphosite.org/homeAction>). Phosphorylation sites on DNAJB6b were listed in the chart. Low-throughput papers (LTP) indicate that the number of records in which this modification site was determined using methods other than discovery mass spectrometry. High-throughput papers (HTP), on the other hand, report the number of records in which this modification site was assigned solely by proteomic mass spectrometry.

**Table S2. The phosphorylation sites on DNAJB6 were reported in the PhosphoNET database.**

| Site | Amino acid sequence of DNAJB6 | Experimentally documented/ confirmed site |
| --- | --- | --- |
| S15 | LGVQRHA(pS)PEDIKKA | PhosphoSitePlus |
| Y23 | PEDIKKA(pY)RKLALKW | PubMed ID 16641100-rat |
| Y53 | FKQVAEA(pY)EVLSDAk | PubMed ID 18083107 |
| Y65 | DAKKRDI(pY)DKYGKEG | PhosphoSitePlus |
| S226 | EEDGQLK(pS)LTINGVA | PhosphoSitePlus |
| T228 | DGQLKSL(pT)INGVADD | PhosphoSitePlus |
| S267 | PKPPRPA(pS)LLRHAPH | PhosphoSitePlus |
| S277 | RHAPHCL(pS)EEEGEQD | PubMed ID 18669648 |

Candidates of the DNAJB6b PTM site are assessed through the available data from PhosphoNET (http://www.phosphonet.ca). Only DNAJB6 phosphorylation sites documented or confirmed in mammals were listed in the chart. Three of the phosphorylation sites were experimentally confirmed in PubMed, and IDs were listed. Others were documented in the PhosphoSitePlus database.

**Table S3. Prediction of possible upstream kinases for DNAJB6b by NetPhos 3.1.**

| Phosphorylation site | Sequence (N→C) | Score | | Predicted kinase | |
| --- | --- | --- | --- | --- | --- |
| Y53 | VAEA**Y**EVLS | 0.555 | unsp | |  |
|  |  | 0.412 | INSR | |  |
|  |  | 0.408 | SRC | |  |
|  |  | 0.358 | EGFR | |  |

Possible upstream kinases responsible for the phosphorylation of DNAJB6b at Y53 are listed in the chart. The closer the probability score is to 1, the more likely it is that the residue is phosphorylated by the predicted kinase. In this case, candidates with scores higher than 0.4 are listed. INSR has a score of 0.412, and SRC has a score of 0.408 for phosphorylating DNAJB6b Y53. NetPhos 3.1 server is available at: <http://www.cbs.dtu.dk/services/NetPhosK/>. (unsp: non-specific prediction)

**Table S4. The primers used for cloning.**

| Primers | Sequence | Source |
| --- | --- | --- |
| DNAJB6-Y53F-BstBI-For | 5’GCAAGTAGCGGAGGCATTCGAAGTGCTGTCGGATGC3’ | IDT |
| DNAJB6b-Y53E-NdeI^del^-For | 5’GCAAGTAGCGGAGGCAGAAGAAGTGCTGTCGGATG3’ | IDT |
| pFLAG-CMV-2-hYES1-XbaI-For | 5’ACCAGTCGACTCTAGAATGGGCTGCATTAAAAGTAA3’ | IDT |
| pFLAG-CMV-2-hYES1-SmaI-Rev | 5’ACAGGGATGCCACCCGGGTTATAAATTTTCTCCTGGCTGGT3’ | IDT |
| pFLAG-CMV-2-hHSPA8 E218A/K220A-EcoRI-For | 5’TTGCGGCCGCGAATTCATC3’ | IDT |
| hHSPA8 E218A/K220A-For | 5’TGcGGTCgcGTCTACAGCTGGAGACAC3’ | IDT |
| hHSPA8 E218A/K220A-Rev | 5’GTAGACgcGACCgCAAAGATTCCATCCTCAATA3’ | IDT |
| pFLAG-CMV-2-hHSPA8 E218A/K220A-EcoRI-Rev | 5’GGACTCAAGTGAATTCTTGG3’ | IDT |

**Table S5. The oligonucleotide sequences of shRNA.**

| Oligonucleotide sequences of shRNA | Source | Identifier |
| --- | --- | --- |
| pLKO.1-shLuc Target seq.: 5’GCGGTTGCCAAGAGGTTCCAT3’ | Academia Sinica | TRCN0000072249 |
| pLKO.5-shLuc Target seq.: 5’ GCGGTTGCCAAGAGGTTCCAT3’ | Academia Sinica | TRCN0000231719 |
| pLKO.1-shSRC #1 Target seq.: 5’ GCTGACAGTTTGTGGCATCTT3’ | Academia Sinica | TRCN0000199313 |
| pLKO.1-shSRC #2 Target seq.: 5’ CATCCTCAGGAACCAACAATT3’ | Academia Sinica | TRCN0000195339 |
| pLKO.1-shFYN #1 Target seq.: 5’ GCGATCAGCAAACATTCTAGT3’ | Academia Sinica | TRCN0000003097 |
| pLKO.1-shFYN #2 Target seq.: 5’ GTGCCAACAATCCTAGTGCTT3’ | Academia Sinica | TRCN0000003101 |
| pLKO.5-shLYN #1 Target seq.: 5’ GGAATCCTCCTATACGAAATT3’ | Academia Sinica | TRCN0000218210 |
| pLKO.5-shLYN #2 Target seq.: 5’ GAGTGACGATGGAGTAGATTT3’ | Academia Sinica | TRCN0000230901 |
| pLKO.1-shYES #1 Target seq.: 5’ CCAAAGTCAGAATTGCTCAAA3’ | Academia Sinica | TRCN0000121242 |
| pLKO.1-shYES #2 Target seq.: 5’ GCTGTCATTATTTCCTCTTAT3’ | Academia Sinica | TRCN0000121062 |

**Table S6. The plasmids used in this study.**

| Plasmid | Source | Identifier |
| --- | --- | --- |
| pEGFP-C1 | Clontech | Cat# 6084-1 |
| pEGFP-C1-tau P301L (2N4R) | (Chang et al., 2023) | N/A |
| VN-tau (P301L) | Addgene | Cat# 87634 |
| tau (P301L)-VC | Addgene | Cat# 87633 |
| pcDNA5/FRT/TO-V5 control | (Chang et al., 2023) | N/A |
| pcDNA5/FRT/TO-V5-DNAJB6b | Addgene | Cat#19528 |
| pcDNA5/FRT/TO-V5-DNAJB6b Y53F | This study | N/A |
| pcDNA5/FRT/TO-V5-DNAJB6b Y53E | This study | N/A |
| pFLAG-CMV-2 control | Sigma | Cat# E7033 |
| pFLAG-CMV-2-hYES1 | This study | N/A |
| pFLAG-CMV-2-hHSPA8 | This study | N/A |
| pFLAG-CMV-2-hHSPA8 E218A/K220A | This study | N/A |
| pET-28a-DNAJB6b | This study | N/A |
| pET-28a-DNAJB6b Y53F | This study | N/A |

**Table S7. The antibodies used in this study.**

| Antibodies | Source | Identifier |
| --- | --- | --- |
| DNAJB6 | Abcam | Cat# ab198995  RRID: AB_2924896 |
| HSPA8 | Novus | Cat# NB120-2788  RRID: AB_2120309 |
| HSPA1A (HSP70 C92F3A-5) | Santa Cruz Biotechnology | Cat# sc-66048  RRID: AB_832518 |
| tau | GeneTex | Cat# GTX112981  RRID: AB_10730753 |
| Phospho-Tau (Ser202, Thr205) Monoclonal Antibody (AT8) | Thermo Fisher Scientific | Cat# MN1020  RRID: AB_223647 |
| V5 | Abcam | Cat# ab9116  RRID: AB_307024 |
| V5 | Cell Signaling Technology | Cat# 13202  RRID: AB_2687461 |
| V5 | Thermo Fisher Scientific | Cat# R960-25  RRID: AB_2556564 |
| β-actin | Proteintech | Cat# 60008-1-Ig RRID: AB_2289225 |
| GFP | GeneTex | Cat# GTX113617  RRID: AB_1950371 |
| FLAG | Sigma-Aldrich | Cat# F3165  RRID: AB_259529 |
| Phospho-AKT (Ser473) | Cell Signaling Technology | Cat# 9271  RRID: AB_329825 |
| AKT | Cell Signaling Technology | Cat# 9272  RRID: AB_329827 |
| Phospho-Src Family (Tyr416) | Cell Signaling Technology | Cat# 2101  RRID: AB_331697 |
| SRC | Cell Signaling Technology | Cat# 2108  RRID: AB_331137 |
| FYN | GeneTex | Cat# GTX109428  RRID: AB_10729465 |
| LYN | GeneTex | Cat# GTX101222  RRID: AB_10720516 |
| YES | Cell Signaling Technology | Cat# 3201  RRID: AB_11178531 |
| Normal Rabbit IgG | Cell Signaling Technology | Cat# 2729 RRID: AB_1031062 |
| Peroxidase AffiniPure® Goat Anti-Rabbit IgG (H+L) | Jackson ImmunoResearch | Cat# 111-035-003 RRID: AB_2313567 |
| Peroxidase AffiniPure® Goat Anti-Mouse IgG (H+L) | Jackson ImmunoResearch | Cat# 115-035-003 RRID: AB_10015289 |
| Alexa Fluor™ 594 Goat anti-Mouse IgG | Invitrogen | Cat# A-11005  RRID: AB_2534073 |

**Table S8. The human brain samples used in this study.**

| Catalog | Lot # | Sex | Age | Ethnicity | Pathology |
| --- | --- | --- | --- | --- | --- |
| NB820-59177 | C511134 | Male | 26 | Asian | Normal clinical diagnosis |
| NB820-59177 | C807591 | Male | 82 | Caucasian | Normal clinical diagnosis |
| NB820-59177 | C910514 | Male | 71 | Caucasian | Normal clinical diagnosis |
| GTX28771 | 822400674 | Male | 82 | Caucasian | Aortic stenosis |
| GTX27918 | 822600376 | Male | 71 | N/A | Normal clinical diagnosis |
| NB820-59363 | C511136 | Female | 93 | Hispanic | Alzheimer's disease, Lymphoma |
| NB820-59363 | C511137 | Male | 87 | Caucasian | Alzheimer's disease, Prostate cancer |
| NB820-59363 | C910505 | Female | 93 | Hispanic | Alzheimer's disease, Lymphoma |
| GTX26622 | 822304853 | Female | 93 | N/A | Alzheimer's disease, Lymphoma |
| GTX26622 | 822502057 | Female | 87 | N/A | Alzheimer's disease |

**Table S9. The chemicals and reagents used in this study.**

| Chemicals and Reagents | Source | Identifier |  |
| --- | --- | --- | --- |
| Dulbecco's Modified Eagle Medium/Nutrient Mixture F-12 (DMEM/F12) medium | Cytiva | Cat# SH30023.02 |  |
| Antibiotic/Antimycotic Solution (100x) | Capricorn Scientific | Cat# AAS-B |  |
| Avantor® Seradigm, Select Grade Fetal Bovine Serum (FBS) | VWR | Cat# 89510-186 |  |
| Puromycin | Sigma-Aldrich | Cat# P8833 |  |
| Dimethyl sulfoxide (DMSO) | Sigma-Aldrich | Cat# D8418-100ML |  |
| NVP-AEW541 | MedChemExpress | Cat# HY-50866 |  |
| Dasatinib | MedChemExpress | Cat# HY-10181 |  |
| CH6953755 | MedChemExpress | Cat# HY-135299 | |
| PP2 | MedChemExpress | Cat# HY-13805 |  |
| RIPA Lysis Buffer, 10X | Merck Millipore | Cat# 20-188 |  |
| cOmplete™ EDTA-free Protease Inhibitor Cocktail | Roche | Cat# 4693132001 |  |
| PhosSTOP™ inhibitor tablets | Roche | Cat# 4906837001 |  |
| Protein G Mag Sepharose Xtra magnetic beads | Cytiva | Cat# 28967070 |  |
| TALON® Superflow™ histidine-tagged protein purification resin | Cytiva | Cat# 28957499 |  |
| Amicon Ultra-0.5 Centrifugal Filter Unit  (3 kDa) | Merck Millipore | Cat# UFC500396 |  |
| Human YES1, GST Tag Recombinant Protein | Thermo Fisher Scientific | Cat# A15557 | |
| PageRuler™ Prestained Protein Ladder  (10-180 kDa) | Thermo Fisher Scientific | Cat# 26616 |  |
| GXBio Prestained Protein Ladder  (25-300 kDa) | Biohelix | Cat# XP25300 |  |
| Luminata™ Crescendo Western HRP Substrate | Merck Millipore | Cat# WBLUR0500 |  |
| Opti-MEM® I Reduced Serum Medium | Gibco | Cat# 31985070 |  |
| Lipofectamine™ LTX reagent with PLUS™ reagent | Thermo Fisher Scientific | Cat# 15338100 |  |
| Bio-Rad Protein Assay | Bio-Rad Laboratories | Cat# 5000006 |  |
| Duolink® In Situ Wash Buffers, Fluorescence | Sigma-Aldrich | Cat# DUO82049 |  |
| Duolink® In Situ PLA® Probe Anti-Mouse PLUS | Sigma-Aldrich | Cat# DUO92001 |  |
| Duolink® In Situ PLA® Probe Anti-Rabbit MINUS | Sigma-Aldrich | Cat# DUO92005 |  |
| Duolink® In Situ Detection Reagents Red | Sigma-Aldrich | Cat# DUO92008 |  |
| DAPI | Thermo Fisher Scientific | Cat# D1306 |  |
| Fluoromount™ Aqueous Mounting Medium | Sigma-Aldrich | Cat# F4680 |  |
| Cover glasses circular, 15 mm Ø No. 1 (0.13-0.16 mm) | Marienfeld Laboratory Glassware | Cat# 0111550 |  |
| Cover glasses square, 22 × 22 mm Ø No. 1 (0.13-0.16 mm) | Marienfeld Laboratory Glassware | Cat# 0101010 | |
| Cellulose Acetate Membrane Filters, 0.2 μm | Sterlitech | Cat# CA029025 | |
| Nitrocellulose Membrane, 0.2μm | Bio-Rad Laboratories | Cat# 1620112 | |

**Table S10.** **The instruments and software used in this study.**

| Instrument and software | Source | Identifier |
| --- | --- | --- |
| ChemiDoc™ Imaging System | Bio-Rad | Cat# 12003153 |
| 96-well dot-blot apparatus | Bio-Rad | Cat# 170-6545 |
| ApoTome.2 microscope | Zeiss | N/A |
| Zen Blue 2.6 | Zeiss | N/A |
| ImageJ | NIH | <https://imagej.net/> |
| Image Lab | Bio-Rad | <https://www.bio-rad.com/en-tw/product/image-lab-software?ID=KRE6P5E8Z> |
| BioRender | N/A | <https://biorender.com/> |
